# Analytically Unsupervised Metabolomic Profile of the Premium Malgasy Pepper Voatsipérifery (*Piper borbonense*): Identification of Marker Components

**DOI:** 10.1021/acs.jafc.5c01501

**Published:** 2025-04-11

**Authors:** Elena Serino, Federica Pollastro, Paolo Luciano, David Touboul, Giovanni Appendino, Giuseppina Chianese, Orazio Taglialatela-Scafati

**Affiliations:** † Department of Pharmacy, School of Medicine and Surgery, University of Naples Federico II, Via D. Montesano 49, 80131 Napoli, Italy; ‡ Department of Pharmaceutical Sciences, University of Piemonte Orientale, 28100 Novara, Italy; § Laboratoire de Chimie Moléculaire (LCM), UMR 9168, CNRS, Ecole Polytechnique, Institut Polytechnique Paris, Palaiseau 91190, France

**Keywords:** *Piper borbonense*, untargeted metabolomics, phytochemical, piperamides, volatilome

## Abstract

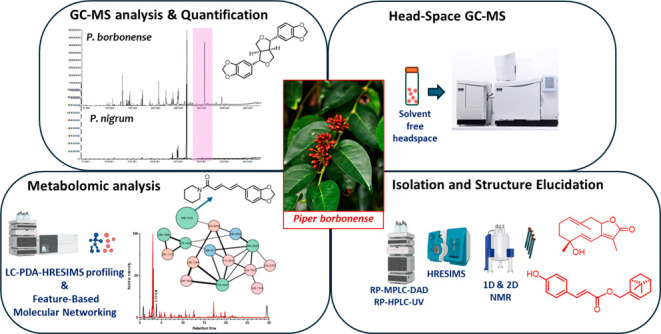

The berries of *Piper borbonense* (Miq.) C. DC., a wild vine native to Madagascar, are prized for their distinctive aroma and flavor, considered superior to the ones of the domesticated peppers (*P. nigrum* L., *P. longum* L.). The scarcity of studies on *P. borbonense*, locally known as voatsipérifery, makes it difficult to secure the identity of its berries and complement its sensory analysis. This supports the need for a comprehensive phytochemical investigation utilizing complementary analytical techniques. Headspace gas chromatography highlighted differences in the “volatilome” of *P. borbonense* and *P. nigrum*, while an untargeted metabolomic analysis, based on the LC-MS^2^-based feature-based molecular networking tool, annotated different classes of compounds (monoterpenoids, sesquiterpenoids, monolignols, lignans, and piperamides). This analysis next guided the isolation of 40 fully characterized compounds, including two new natural products [the sesquiterpene lactone 4-hydroxyisogelehomanolide (**29**) and the hydroxycinnamate ester borbonensin (**38**)]. The phytochemical profile of voatsipérifery is remarkable for the presence of the nonvolatile monoterpene *p*-menth-5-en-1,2-diol (**23**), of sesquiterpene lactones, and of large amounts of sesamin (**34**), a marker trait that clearly distinguishes it from those of the cultivated peppers of commerce and was confirmed with a parallel investigation of *P. nigrum*. Overall, our study underlines the relevance of advanced metabolomic approaches to characterize the phytochemical profile of spices and identify specific marker compounds.

## 1 Introduction

The genus *Piper* L., one of the largest in the Piperaceae family, includes more than 700 species endemic to tropical and subtropical regions, with Southeast Asia and South/Central America being its main hubs of diversity. The economic relevance of *Piper* plants, as exemplified by black- (*P. nigrum* L.) and long- (*P. longum* L.) peppers, can hardly be underestimated: the search for peppers fueled the Age of Exploration, and black and long peppers are still some of the most widely used spices in the world.^1^

*P. borbonense* (Miq.) C. DC. is a rare Macaronesian species, whose berries are locally known as *voatsipérifery*, a word derived from the Malagasy *voa* (“fruit”) and *tsiperifery* (literally “which makes sores not exist”). The *voatsipérifery* vine can grow up to 15 m on large trees of the tropical forests of Réunion, Mauritius, and Madagascar, where it is traditionally collected. The plant is harvested entirely by hand by Malagasy communities, whose villagers climb the high vines to collect the fresh berries. The rarity of this *Piper* and the manual labor involved in its collection justify its high market price, about $ 200 per kilogram, at least 1 order of magnitude higher than that of *P. nigrum*.^2^ Over the past decades, *P. borbonense* has become very popular in gourmet cuisine, where it is valued for its distinctively delicate flavor, characterized by lower pungency and more scents compared to those of other peppers.^3^ This “*best pepper in the world*” (as qualified by the triple Michelin-starred chef Anne-Sophie Pic)^4^ is commonly added at the end of meat cooking to preserve its aroma, but it has also been associated with fish, fruits, and other foods. Because of its growing popularity and price, *P. borbonense* has been adulterated with the cultivated peppers of commerce as well as with related Malagasy wild peppers, like *P. malgassicum* and *P. tsarasotrae*, which belong to the same clade.^5^ Unambiguous species identification and authentication remain challenging, but the combination of botanical features along with the composition of its essential oil and the piperine contents has been proposed as a combined system to validate its identity, even though differences with the cultivated peppers are associated with specific ratios of constituents and not with marker compounds.^6^

*P. borbonense* has also been used in folk medicine for a host of conditions (diarrhea, diabetes, malaria, worm infections, dental health)^7^ that have inspired recent scientific evaluations of its medicinal properties. Thus, a mixture of phenolics from *P. borbonense* was evaluated for immunomodulatory activity,^7^ and the essential oils for fungicidal activity.^8^

The composition of the essential oil has been recently investigated, highlighting the high aromatic potential of *P. borbonense*, which contains 9.8% essential oil and exhibits a monoterpene-rich profile, with limonene as the predominant contributor to its characteristic aroma.^2^ On the other hand, *P. borbonense* has a significantly lower piperine content than black pepper. Additionally, an analysis of its major piperamides has been conducted, comparing its profile with that of other *Piper* species, and evidencing a distinct composition, with some qualitative and quantitative differences.^9^ However, a comprehensive analysis of the secondary metabolites from *P. borbonense* is still lacking, and specific markers are yet to be identified. To fill this gap, we have carried out an untargeted metabolomic analysis of *voatsipérifery* using an LC-MS^2^-based molecular networking. This is a powerful tool in food metabolomics that enables analysis, identification, and annotation of components, revealing the chemical diversity of food composition. Interpreting the food metabolome helps uncover biomarkers associated with food safety, function, and quality.^10,11^ Moreover, starting from this untargeted dereplication step, we carried out isolation and subsequent structural characterization of 40 constituents, using 1D and 2D NMR, two of which are new natural products.

## 2 Materials and Methods

### 2.1 Plant Material and Extraction

*Piper borbonense* fruits (origin Madagascar) were purchased from A. Minardi & Figli, Bagnacavallo (RA). A voucher sample is stored in Novara Laboratory at the Department of Pharmaceutical Sciences (code PB-19). Organic black piper (*Piper nigrum*) in grain was purchased from the Biojoy GmbH company (Germany). A voucher sample is stored at the Department of Pharmacy, University of Naples Federico II (n. 2024/023).

Extraction with acetone (3 × 5 L) of the berries (337.5 g) of *P. borbonense* provided, after evaporation, 53.5 g (15.88%) of a dark gum, used as such for all studies.

### 2.2 General Experimental Procedures

UV and Electronic Circular Dichroism (ECD) spectra were registered on a JASCO J-710 instrument. Solvents for extraction and chromatographic purifications (i.e., acetonitrile, MeOH, and water) were HPLC grade, and UPLC/MS-grade solvents for sample preparation and LC-MS/MS analysis were purchased from Merck Life Science S.r.l. Chemicals and solvents were used without any further purification unless stated otherwise. Chromatographic purifications were carried out using MPLC (Medium Pressure Liquid Chromatography) and HPLC (High Pressure Liquid Chromatography). MPLC-DAD separations were performed on an Interchim instrument, puriFlash XS 520 Plus (Sepachrom s.r.l., Milan, Italy). HPLC-UV–vis separations were performed on an Agilent instrument, using a 1260 Quat Pump VL system, equipped with a 1260 VWD VL UV–vis detector and a Knauer 1800 apparatus equipped with a refractive index detector and a Rheodyne injector. ^1^H (400 and 600 MHz) and ^13^C (100 and 150 MHz) NMR spectra were measured on a Bruker spectrometer. Chemical shifts are referenced to the residual solvent signal (CDCl_3_: δ_H_ 7.26, δ_C_ 77.0; CD_3_OD: δ_H_ 3.31, δ_C_ 49.3). Homonuclear ^1^H connectivities were determined by COSY (COrrelation SpectroscopY) experiments. Through-space ^1^H connectivities were evidenced using a NOESY (Nuclear Overhauser Enhancement SpectroscopY) experiment with a mixing time of 300 ms. One-bond heteronuclear ^1^H–^13^C connectivities were determined by the HSQC (Heteronuclear Single Quantum Correlation) experiment; two- and three-bond ^1^H–^13^C connectivities by gradient-HMBC (Heteronuclear Multiple Bond Correlation) experiments optimized for a ^2,3^*J* of 8 Hz. HR-ESIMS experiments were performed on an LTQ-Orbitrap mass spectrometer controlled by Excalibur data system. Optical rotations (CHCl_3_ and MeOH) were measured at 589 nm on a JASCO P2000 polarimeter. ECD spectra were obtained on a Jasco J-815 CD spectropolarimeter (Jasco, Mary’s Court, Easton, MD, USA) with a 0.1 mm cuvette and four accumulations. LC-MS/MS analysis was performed on an LTQ-XL Ion Trap mass spectrometer equipped with an Ultimate 3000 HPLC or on an LTQ Orbitrap XL FT-MS mass spectrometer equipped with an Ultimate 3000 HPLC. All the LC-MS instruments were from Thermo Fisher Scientific Spa, Rodano, Italy. Chromatographic separation was achieved on a Kinetex 2.6 μm polar C18 100 Å (100 × 3 mm) column (Phenomenex).

### 2.3 Head-Space Gas Chromatography

Head-space gas-chromatography analysis was carried out on a PerkinElmer Clarus 600 gas chromatograph equipped with a PerkinElmer Clarus 600 mass spectrometer and a PerkinElmer TurboMatrix 40 headspace sampler; 120.0 mg of the sample (*P. borbonense* fruits) was crushed and inserted in a sealed vial, which was heated in the sampler at 80 °C for 10 min before the injection. Helium was used as a gas carrier, and 1 μL was injected into the transfer line at 150 °C and 30 psi. The VOCs were carried by He through the transfer line maintained at 180 °C for analysis in the GCMS system. The gas chromatograph was equipped with a DB-5 column (60 m × 0.25 mm × 0.25 μm inner diameter). The oven temperature was increased from 50 to 240 °C at 6 °C/min, to 310 °C at 10 °C/min, holding for 10 min, resulting in 46 min of measurement time. The total column pressure was kept constant at 26 psi in the splitless mode. The mass spectra were obtained operating in electronic ionization mode (EI) at 70 eV, and the ion source temperature was set at 200 °C, with a scan mass range of *m*/*z* 40–500 and a sampling rate of 0.2 s. Peak identification was accomplished by comparison of their mass spectra and fragmentation patterns with those stored in the NIST database.

### 2.4 LC-MS/MS Analysis and Molecular Networking

The acetone extract of *P. borbonense* was subjected to an LC-MS/MS analysis on an LTQ Orbitrap XL FT-MS mass spectrometer (Thermo Fisher Scientific Spa, Rodano, Italy) equipped with an Ultimate 3000 HPLC (Thermo Fisher Scientific Spa, Rodano, Italy). The chromatographic separation was carried out on a Kinetex 2.6 μm polar C18 100 Å (100 × 3 mm) column (Phenomenex). The LC-MS/MS experiment was carried out using a combination of 0.1% (v/v) formic acid in H_2_O (A) and MeCN (B) as the mobile phase; the gradient elution was optimized as follows: 50% B for 3 min, 50–95% B over 20 min, held 10 min before returning to the initial conditions. The total run time, including the column wash and the equilibration, was 30 min, flow rate 0.5 mL/min, injection volume 5 μL. The MS and MSn spectra, in positive mode, were recorded in data-dependent acquisition mode, inducing fragmentation of the five most intense peaks for each scan. Source conditions were as follows: spray voltage, 3.5 kV (positive mode); capillary voltage, 25 V; source temperature, 320 °C; normalized collision energy, 25. The acquisition range was *m/z* 150–1500. The LC-MSMS data were converted from .raw to .mzML using MSConvert part of the ProteoWizard 3.0 package before being processed on Mzmine 2.53 software using the following parameters: mass detection noise level 1000 for mass level 1; mass detection noise level was set as 50 for nose level 2; the ADAP chromatogram builder was used with a minimum of 5 consecutive scans with a minimum intensity of 1000 and a minimum absolute height of 1000, the *m*/*z* tolerance was set at 0.005 or 15 ppm; deconvolution was performed with the ADAP resolver with a signal-to-noise ratio of 30, a minimum feature height of 1000, a coefficient/area threshold set at 10, a peak duration range of 0.0–1.0 min and the RT wavelet range set between 0.01 and 0.10; the isotope peak grouper was used with the *m*/*z* tolerance set at 0.005 or 15 ppm, an RT tolerance of 0.1 min and the maximum number of charges set at 2. The molecular network was generated on MetGem open source software (Version 1.5.2), setting *m/z* tolerance 0.04, minimum matched peaks 3, keeping peaks outside ± 17 Th, keeping peaks above 1%, and in the top 6 in the 50 Th window. Then, the maximum neighbor number was set at 10, the minimum score value at 0.50, and max connected component size at 1000. The molecular networks were visualized using the Cytoscape_v3.7.2 software.3.

### 2.5 GC-MS Analysis and Quantitative Analysis

GC–MS analysis was performed on an Agilent 6850 Ser. II apparatus, fitted with a fused silica HP-5 capillary column (30 m x 0.25 mm), 0.33 mm film thickness, coupled to an Agilent Mass Selective Detector MSD 5973; ionization voltage 70 eV; electron multiplier energy 2000 V. Helium was used as the carrier gas (1 mL/min). Gas chromatographic conditions were: column temperature at 40 °C, with 5 min initial hold, and then to 260 °C at 2 °C min^–1^, 260 °C (20 min). Interface temperature 295 °C; mass range was *m*/*z* 29–600, ionization energy 70 eV, multiplier energy 2000 V, scan time 1 s. Peak identification was accomplished by comparison of their mass spectra with those stored on the GC–MS databases (NIST 02 and Wiley 275) and reported in the literature.

### 2.6 Isolation of Pure Compounds

An aliquot of the acetone extract of *P. borbonense* (4.7 g) was purified on MPLC-DAD on a silica cartridge 60 Å 50 μ-size 120 (180 g)–column volume (CV) 153 mL. The mobile phase was 0.1% (v/v) formic acid in H_2_O (A), MeCN (B), and methanol (C) with the following gradient method: starting conditions: 80% A–20% B for CV 0–2; 70% A–30% B for CV 4–6; 60% A–40% B for CV 8–10; 50% A–50% B for CV 13–16; 40% A–60% B for CV 18–20; 10% A–90% B for CV 30–32; 5% A–95% C for CV 33–35; 100% C for CV 37–42; the flow rate was 40.0 mL/min and the UV detection was set at 270 nm. This separation afforded 26 fractions (A–Z). Fraction B (73.1 mg) was identified as 5-*p*-menthene-1,2-diol (**23**). Fraction D (18.7 mg) was further purified on RI-HPLC using a Luna 5 μm C18 100 Å 250 × 4.6 mm, flow rate 1.0 mL/min, using the isocratic elution 50% A-50% C, affording compound **24** (0.8 mg). Fraction E (26.9 mg) was further purified on RP-HPLC using a mixture of 55% A-45% C as the mobile phase on a Luna 5 μm C18 100 Å 250 × 4.6 mm, with a flow rate of 1.0 mL/min. This yielded compounds **30** (0.8 mg, Rt 12.4 min), **25** (0.6 mg, Rt 0.6 min), **26** (0.5 mg, Rt 33.4 min), the new **29** (1.4 mg, Rt 40.0 min), and **27** (1.0 mg, Rt 43.1 min). Fraction G (43.4 mg) was purified on an HPLC-UV, setting the UV detection wavelength at 270 nm, using a Luna 10 μm C18 100 Å 250 × 10 mm, flow rate 3.0 mL/min, to give **31** (1.5 mg, Rt 17.6 min), **32** (1.2 mg, Rt 19.2 min), **1** (2.3 mg, Rt 24.4 min) and **4** (10.3 mg, Rt 27.7 min). Fraction H (16.5 mg) was purified by HPLC-UV, setting the UV detection at 230 nm, with a Luna 5 μm C18 100 Å 250 × 4.6 mm, flow rate 1.0 mL/min, and a mixture of 50% A-50% C as the mobile phase. Compounds **17** (0.4 mg, Rt 16.7 min), **33** (0.5 mg, Rt 16.7 min), **36** (0.3 mg, Rt 21.7 min), **2** (2.3 mg, Rt 23.4 min), **5** (0.6 mg, Rt 26.3 min) and **3** (1.0 mg, Rt 28.4 min) were isolated. Piperine (**6**) was obtained from fraction I (495.6 mg) as a white powder. Fraction J (29.4 mg) was purified by HPLC-UV, with UV detection wavelength set at 270 nm, a Luna 5 μm C18 100 Å 250 × 4.6 mm, a flow rate of 1.0 mL/min, and an isocratic elution of 35% A–65% C, yielding to **7** (0.5 mg, Rt 15.0 min), **35** (3.2 mg, Rt 17.1 min), **9** (0.8 mg, Rt 21.1 min), and **8** (0.7 mg, Rt 27.1 min). Sesamine was obtained in pure form from fraction K (221.8 mg). Fraction L (92.7 mg) was purified by MPLC using a Silica Cartridge 100Å 25 μ-size 4 (5.4 g). The column volume (CV) was 5 mL, with UV wavelength set at 270 nm and at a 5 mL/min flow rate. The following gradient was used: 30% A - 70% C for CV 0–5, 25% A–75% C for CV 15–25; 20% A–80% C for CV 35–45; 15% A–85% C for CV 55–60; 5% A–95% C for CV 65–70. This yielded compounds **28** (14.0 mg, CV 9–11) and **18** (13.6 mg, CV 13–15). Fraction M (27.3 mg) was purified by HPLC-UV, setting the UV at 270 nm, with a Luna 10 μm C18 100 Å 250 × 10 mm column, and a flow rate 3.0 mL/min, using as mobile phase 25% A-75% C, obtaining compound **12** (8.8 mg, Rt 28.9 min). Fraction N (38.0 mg) was purified by HPLC-UV using a Luna 5 μm C18 100 Å 250 × 4.6 mm, a flow rate 1.0 mL/min, UV wavelength set at 270 nm, using 25% A-75% C, affording **19** (3.6 mg, Rt 16.3 min), **10** (0.5 mg, Rt 20.3 min), **13** (1.9 mg, Rt 25.8 min). Fraction O (57.8 mg) was purified by HPLC-UV, setting UV at 300 nm, using a Luna 5 μm C18 100 Å 250 × 4.6 mm, with a flow rate 1.0 mL/min, and an isocratic method 20% A-80% C. Compounds **14** (1.8 mg, Rt 17.9 min) and the novel **38** (1.0 mg, Rt 18.8 min) were isolated. Fraction P (80.6 mg) was purified by MPLC using a silica cartridge 100Å 25 μ - size 4 (5.4 g)–column volume (CV) 5 mL, setting the UV wavelength at 270 nm, flow rate 5 mL/min, and using the following gradient system: 50% A–50% C for CV 0–5, 20% A–80% C for CV 55–60; 5% A–95% C for CV 65–67. This yielded **37** (8.9 mg, CV 16–19) and **15** (6.4 mg, CV 20–23). Fraction Q (29.7 mg) was purified by HPLC-UV equipped with a Luna 10 μm C18 100 Å 250 × 10 mm column, with a flow rate of 3.0 mL/min, and the isocratic method 15% A–85% C, with UV wavelength set at 270 nm. This afforded **11** (5.8 mg, Rt 20.6 min) and **16** (10.5 mg, Rt 24.8 min). Fraction X (77.3 mg) was purified by MPLC-DAD using a Silica Cartridge 60 Å 50 μ - size 4 (4.0 g)–column volume (CV) 5 mL and the following gradient: hexane (A) EtOAc (C) 90% A–10% C for CV 0–5; 80% A–20% C for CV 10–15, 70% A–30% C for CV 20–25, setting UV wavelength at 220 and 270 nm and with 10 mL/min as flow rate. This yielded compounds **20** (10.5 mg, CV 3–4) and **22** (3.8 mg, 5 CV). Fraction Y (98.9 mg) was purified by MPLC a Silica Cartridge 60Å 50 μ - size 12 (12.0 g)–column volume (CV) 25 mL and using the following gradient method hexane (A), EtOAc (C) as the mobile phase with 100% A for CV 0–5; 90% A–10% C for CV 10–15 and 80% A–20% C for CV 20–25, with 15 mL/min as flow rate, yielding compound **21** (20.6 mg, CV 17–19).

*E,E-*4-Hydroxygermacra-1(10),5,7(11)-trien-8-olide (**29**): yellowish amorphous solid, [α]*_D_^25^* + 99 (*c* 0.12, MeOH); HRESIMS [M + H]^+^*m*/*z* found 249.1489 (C_15_H_21_O_3_ requires 249.1485). ^1^H NMR (CDCl_3_, 600 MHz) 6.11 (1H, d, *J* = 17.0 Hz, H-6), 5.89 (1H, d, *J* = 17.0 Hz H-5), 5.11 (1H, brd, *J* = 11.4 Hz, H-8), 4.94 (1H, brd, *J* = 10.6 Hz, H-1), 2.90 (1H, brd, *J* = 11.9 Hz, H-10a), 2.63 (1H, m, H-2a), 2.03 (2H, overlapped, H-2b, H-3a), 2.02 (1H, overlapped, H-10b), 1.88 (3H, s, H-13), 1.81 (3H, s, H-14), 1.77 (1H, overlapped, H-3b), 1.48 (3H, s, H-15); ^13^C NMR (CDCl_3_, 150 MHz) 174.9 (C-12), 159.2 (C-7), 152.9 (C-5), 139.6 (C-1), 127.1 (C-10), 121.6 (C-11), 116.3 (C-6), 77.8 (C-8), 75.5 (C-4), 49.3 (C-3), 46.1 (C-9), 27.5 (C-15), 23.9 (C-2), 16.9 (C-14), 8.8 (C-13).

Borbonensin (**38**): white amorphous solid, [α]_D_^25^ + 6.8 (*c* 0.12, MeOH); ESIMS [M-H]^−^*m*/*z* found 297.1490 (C_19_H_21_O_3_ requires 297.1485). ^1^H NMR (CDCl_3_, 600 MHz) 7.64 (1H, d, *J* = 15.9 Hz, H-3), 7.43 (2H, d, *J* = 8.6 Hz, H-5, H-9), 6.83 (2H, d, *J* = 8.6 Hz, H-6, H-8), 6.30 (1H, d, *J* = 15.9 Hz, H-2), 5.58 (1H, m, H-4′), 4.59 (1H, d, *J* = 12.7, 1.3 Hz, H-2′a), 4.55 (1H, dd, *J* = 12.7, 1.3 Hz, H-2′b), 2.41 (1H, m, H-7′a), 2.33 (1H, m, H-5′a), 2.26 (1H, m, H-5′b), 2.17 (1H, dt, *J* = 5.5, 1.2 Hz, H-8′), 2.11 (1H, m, H-6′), 1.30 (3H, s, H-10′), 1.21 (1H, overlapped, H-7′b), 0.85 (3H, s, H-11′); ^13^C NMR (CDCl_3_, 150 MHz) 167.3 (C-1), 157.4 (C-7), 144.3 (C-3), 143.1 (C-3′), 130.1 (C-5, C-9), 127.4 (C-4), 121.1 (C-4′), 115.9 (C-6, C-8), 115.8 (C-2), 66.9 (C-2′), 43.6 (C-8′), 40.6 (C-6′), 38.8 (C-7′), 31.2 (C-5′), 27.1 (C-9′), 26.0 (C-10′), 21.1 (C-11′).

### 2.7 Computational Details

A preliminary conformational search for **29** was conducted using simulated annealing in the INSIGHT II package. The solution phase in MeOH was simulated by setting the corresponding dielectric constant. Conformational energy minimization was achieved through steepest descent, followed by the quasi-Newton–Raphson method (VA09A). Restrained simulations were run for 500 ps using the CVFF force field, as implemented in Discover software (Accelrys, San Diego, CA). The simulation began at 1000 K, and the temperature was gradually decreased to 300 K. A final energy minimization step was performed to refine the obtained structures, using successive applications of steepest descent and quasi-Newton–Raphson (VA09A) algorithms. Both dynamic and mechanical calculations were conducted with flat-well distance restraints set to 1 (kcal/mol)/Å^2^. A total of one hundred structures were generated.

DFT calculations were performed on an Intel(R) Core (TM) i5–4440 processor at 3.0 GHz using the Gaussian16W package (Multiprocessor). GIAO ^13^C NMR calculations were performed using the mPW1PW91 functional and 6–31G(d,p) basis set using the geometry previously optimized at the mPW1PW91/6–31G(d) level as input. DP4+ probabilities were computed using the available DP4+ Toolbox (Excel file). For these calculations, the IEF-PCM solvent continuum model, as implemented in Gaussian (MeOH solvent). The rotatory strength values for electronic transitions from the ground state to singly excited states for the two conformers of **29** were calculated using TDDFT at the M06–2*X*/6–311G(2d,2p) level with Gaussian 16W, including at least 30 excited states in each case and using the IEF-PCM model for MeOH. The rotatory strength values were summed after a Boltzmann statistical weighting, and Δε values were calculated by forming sums of Gaussian functions centered at the wavelengths of the respective electronic transitions and multiplied by the corresponding rotatory strengths. The resulting ECD spectra were then compared to the experimental spectra using SpecDis 1.71 software.

## 3 Results and Discussion

### 3.1 Analysis of the Volatile Compounds of *P. borbonense* Fruits

Headspace gas chromatography–mass spectrometry (HS-GC-MS) has not yet been used to analyze the aroma composition of *P. borbonense*, whose essential oils had been previously investigated only via GC-MS.^2^ Since it requires no sample preparation and can be carried out directly on the crushed berries of the plant, HS-GCMS has the potential to provide a more accurate profile of the volatile components of this highly prized pepper. To highlight the main differences between *P. borbonense* and the common *P. nigrum*, we carried out a comparative analysis of the HS-GC-MS profile of the two species, identifying the compounds using the NIST library (Table 1). In agreement with GC-MS results,^2^ the most abundant compounds were monoterpenes (98.15% in *P. borbonense*, and 97.67% in *P. nigrum*) and, among them, the most abundant compound was δ-3-carene (40.91% in *P. borbonense* and 50.23% in *P. nigrum*). The other major peaks were assigned to d-limonene and α-pinene. The β-pinene content was 10.48% in *P. borbonense* and 12.56% in *P. nigrum*, while β-thujene reached 7.51% in *P. borbonense* and only 0.53% in *P. nigrum*. The total sesquiterpenoid composition was higher in *P. nigrum* (2.15% compared to 1.43% of *P. borbonense*), but, interestingly, *P. borbonense* showed a more diverse composition, since in *P. nigrum* the sesquiterpenoid fraction appears to be dominated by caryophyllene (1.87%, only 0.44% in *P. borbonense*). Finally, phenylpropanoids were not detected in *P. nigrum*, whereas they were found in small amounts (0.32%) in *P. borbonense*. While failing to identify specific markers, the headspace analysis showed an overall distinct profile of volatiles in *P. borbonense* and *P. nigrum*, supporting the claim of a distinct aroma for the two peppers.^4^ Interestingly, some monoterpenes like sabinene and β-thujene (pungent, spicy, woody, and peppery), *p*-cymene (mild, sweet, citrus-like), and α-pinene (fresh, pine-like),^12,13^ show a significantly higher abundance in voatsipérifery, and they can contribute to the definition of the peculiar aroma of this pepper. However, to determine how the terpene profile influences the overall sensory perception of *Piper* species, a sensory evaluation by trained panels is necessary to assess attributes such as aroma, flavor, and pungency.^14^

**Table 1:** Comparison of HS-GC-MS Profiles of *P. borbonense* and *P. nigrum* (Major Compounds Are Highlighted in Bold)

class	compound name	Rt (min)	*P. borbonense* relative abundance^a^	*P. nigrum* relative abundance^a^
Monoterp.	α -phellandrene	12.10	0.56	0.18
Monoterp.	**α-pinene**	12.47	**12.21**	**9.73**
Monoterp.	bicyclo[2.2.1]heptane,7,7-dimethyl-2-methylene-	13.02	n.d.	0.02
Monoterp.	camphene	13.12	0.19	0.11
Monoterp.	**sabinene**	13.68	**1.33**	**0.20**
Monoterp.	1,3,5-cycloheptatriene, 3,7,7-trimethyl-	13.76	0.03	0.03
Monoterp.	**β-myrcene**	13.92	**1.66**	**2.74**
Monoterp.	**β-pinene**	14.00	**10.48**	**12.56**
Monoterp.	α-terpinolene	14.46	0.01	0.03
Monoterp.	**δ-3-carene**	**14.80**	**40.91**	**50.23**
Monoterp.	1,3-cyclohexadiene, 1-methyl-4-(1-methylethyl)-	15.02	0.07	0.09
Monoterp.	o-cymene	15.13	0.04	0.09
Monoterp.	cyclohexene, 1-methyl-5-(1-methylethenyl)-	15.24	0.01	0.08
Monoterp.	**p-cymene**	15.35	**5.52**	**1.86**
Monoterp.	**D-limonene**	15.43	**15.91**	**17.81**
Monoterp.	**β-thujene**	15.58	**7.51**	**0.53**
Monoterp.	eucalyptol	15.66	0.59	0.11
Monoterp.	trans-thujene	16.04	0.01	0.06
Monoterp.	γ-terpinene	16.25	0.10	0.14
Monoterp.	trans-4-thujanol	16.76	<0.01	<0.01
Monoterp.	isoterpinolene	16.91	0.05	0.32
Monoterp.	α-terpinolene	17.08	0.64	0.70
Monoterp.	linalool	17.32	0.09	0.05
Monoterp.	trans-sabinenehydrate	17.75	0.02	nd
Monoterp.	cosmene	18.04	nd	<0.01
Monoterp.	(+)-4-carene	18.43	0.01	nd
Monoterp.	p-mentha-1,5,8-triene	18.76	<0.01	<0.01
Monoterp.	(+)-2-bornanone	19.48	0.01	nd
Monoterp.	p-mentha-1,5-dien-8-ol	19.60	0.01	nd
Monoterp.	trans-sabinenehydrate	20.10	0.01	nd
Monoterp.	benzenemethanol, α,α,4-trimethyl-	20.29	0.01	nd
Monoterp.	α -terpinol	20.50	0.01	nd
Monoterp.	trans-sabinol	20.80	0.07	nd
Monoterp.	linalyl acetate	21.23	0.05	nd
Monoterp.	isopiperitone	22.27	0.02	nd
Phenylprop.	safrolene	23.22	0.15	nd
Sesquiterp.	δ-elemene	23.72	0.02	0.01
Monoterp.	camphenol	23.96	<0.01	nd
Sesquiterp.	α -cubebene	24.05	0.02	<0.01
Sesquiterp.	trans-α-copaene	24.77	0.07	nd
Sesquiterp.	copaene	24.95	0.11	0.13
Sesquiterp.	elemene	25.15	0.07	0.03
Phenylprop.	methyleugenol	25.54	0.11	nd
Sesquiterp.	eremophylene	25.70	nd	0.01
Sesquiterp.	ylangene	26.08	0.01	nd
Sesquiterp.	**caryophyllene**	26.22	**0.44**	**1.87**
Sesquiterp.	α -guaiene	26.30	0.01	nd
Sesquiterp.	copaen-4-α-ol	26.40	0.05	0.01
Sesquiterp.	muurola-3,5-diene	26.83	0.01	nd
Sesquiterp.	humulene	27.10	0.01	0.05
Diterp.	geranyl-α-terpinene	27.21	0.01	nd
Sesquiterp.	γ-muurolene	27.33	0.02	nd
Sesquiterp.	δ-curcumene	27.50	0.02	nd
Sesquiterp.	germacrene D	27.63	0.13	nd
Sesquiterp.	α-guaiene	27.70	0.22	nd
Sesquiterp.	β-bisabolene	27.78	0.07	nd
Sesquiterp.	isoledene	27.82	nd	<0.01
Sesquiterp.	epi-α-selinene	27.92	0.03	0.03
Sesquiterp.	δ-guaiene	27.99	0.03	nd
Phenylprop.	myristicine	28.08	0.01	nd
Sesquiterp.	7-epi-α-cadinene	28.25	0.03	0.01
Sesquiterp.	4-epi-cubebol	28.37	0.02	nd
Sesquiterp.	trans-calamenene	28.51	0.01	nd
Phenylprop.	trans-isomyristicin	28.67	0.02	nd
Phenylprop.	elemicin	28.78	0.03	nd

a Calculated as the area percentage in the total ion chromatogram.

### 3.2 Metabolomic Fingerprint of *P. borbonense* Fruits through LC-UV-MS/MS and FB-MN Analyses

**Figure 1:**
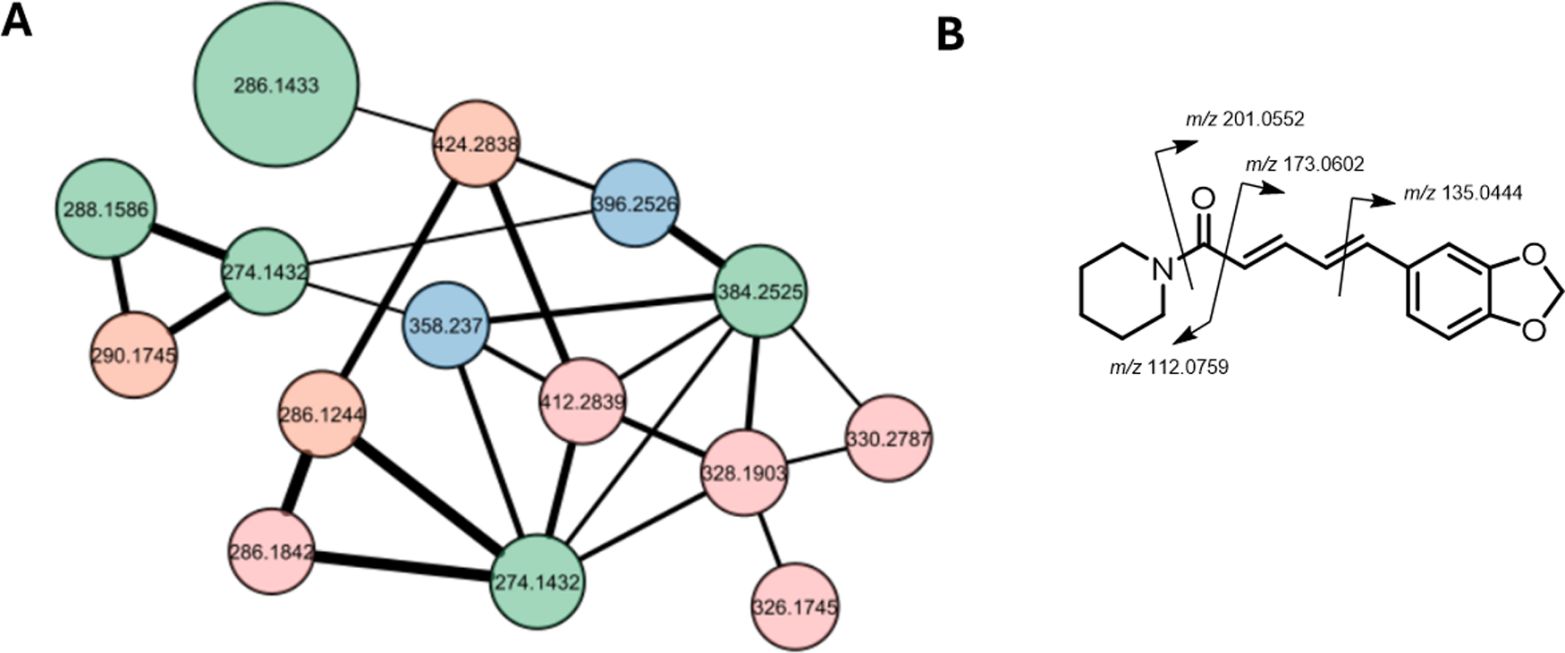
(A) Molecular networking cluster of piperamides. Nodes are labeled with the precursor *m*/*z* value and sized according to the peak area; edge thickness is related to the cosine similarity score. Piperamide nodes were dereplicated and colored green for isolated metabolites and blue for putatively known derivatives. (B) Chemical structure of piperine (*m*/*z* 286.1433) and its diagnostic fragments.

The alkylamide composition of *P. borbonense* has been roughly compared with that of other *Piper* species,^9^ but a comprehensive phytochemical characterization has not yet been carried out. A metabolomic fingerprint of *P. borbonense* was obtained through a preliminary analysis of the crude acetone extract by LC coupled to HR-MS and feature-based molecular network (FB-MN) analysis^15,16^ using MZmine^17^ and MetGem open-source software.^18^ The first software is used to preprocess the raw files, while MetGem compares MS/MS data, immediately visualizing the similarities among the spectra, which are then clustered according to their fragmentation pattern analogies. One of the nodes obtained in the cluster (Figure 1) was assigned to piperine (**6**), precursor ion at *m*/*z* 286.1433, which appears to be the main component of the mixture from the FB-MN and LC-UV-MS^2^ analysis.

The detected fragment ions correspond to the loss of the piperidinyl moiety (*m*/*z* 201.0552), the α-cleavage to the amide group (*m*/*z* 173.0602) and the related fragment with the opposite charge retention (*m*/*z* 112.0759), and finally the loss of the benzodioxole moiety (*m*/*z* 135.0444), in agreement with the data reported in the literature.^9^ Considering the recurrence of the fragment at *m*/*z* 135.0444 in the cluster, we can also assume that the other nodes belong to benzodioxole piperamides. Consequently, they can be assigned to guineesine (**16**) (*m*/*z* 384.2525), clustered with *m*/*z* 396.2526, tentatively identified as 13-(1,3-benzodioxol-5-yl)-1-(1-piperidinyl) and to piperchambamide D (*m*/*z* 358.2371). This latter clustered together with two isomers with different retention time and size (nodes at *m*/*z* 274.1432), one assigned to 4,5-dihydropiperyline (**1**), which closely clustered with piperanine (**3**) (*m*/*z* 288.1586), and the other confidently assigned to piperlonguminin (**5**) given the different fragmentation pattern.

This preliminary information helped a detailed manual analysis of the LC-HRMSMS, resulting in the putative annotation of 36 metabolites (Table 2), based on their molecular formula and fragmentation pattern analysis, that matched those reported in the literature.^9^

**Table 2:** Annotated Compounds from LC-HRMS/MS; Compounds Are Listed in the Order of Elution

compound name	molecular formula	*t*_R_ (min)	precursor ion (*m*/*z*)	Δ (ppm)	fragments in *m*/*z* (peak relative intensity)
4,5-dihydropiperyline (**1**)	C_16_H_19_NO_3_	2.03	274.1430	–2.663	203.0707 (30); 175.0758 (10); 161.0601 (20); 135.0443 (100)
piperyline (**4**)	C_16_H_17_NO_3_	2.14	272.1276	–2.058	201.0551 (100); 173.0602 (5); 143.0494 (2); 135.0443 (10)
dihydropiperlonguminine (**2**)	C_16_H_21_NO_3_	2.58	276.1590	–1.630	203.0704 (70); 175.0757 (10); 161.0599 (20); 135.0441 (100)
piperlonguminin (**5**)	C_16_H_19_NO_3_	2.69	274.1432	–1.897	201.0553 (100); 175.0760 (5); 145.0651; 135.0444 (20)
piperanine (**3**)	C_17_H_21_NO_3_	2.81	288.1586	–2.950	203.0709 (30); 175.0761 (10); 161.0602 (20); 135.0444 (100)
piperine (**6**)	C_17_H_19_NO_3_	3.03	286.1433	–1.5030	201.0552 (100); 173.0602 (5); 135.0444 (10); 112.0759 (5)
7-(1,3-benzodioxol-5-yl)-1-(1-pyrrolidinyl)-2,4-heptadien-1-one	C_18_H_21_O_3_N	3.20	300.1590	–1.499	229.0863 (100); 201.0914 (40); 135.0444 (50)
piperdardine (**8**)	C_19_H_23_NO_3_	4.75	314.1742	–2.706	229.0867 (100); 201.0915 (40); 135.0443 (50)
sarmentine	C_14_H_23_NO	4.98	222.1849	–1.624	151.1120 (100); 133.1014 (50); 124.0759 (20); 95.0492 (70)
marginatine	C_15_H_20_O_2_	5.10	233.1533	–1.357	215.1438 (90); 187.1487 (100); 177.0916 (20); 133.1015 (10)
pellitorine (**18**)	C_14_H_25_NO	6.00	224.2004	–2.235	168.1358 (100); 151.1119 (60); 133.1013 (20); 109.0648 (10)
neopellitorine B (**19**)	C_15_H_25_NO	7.23	236.2004	–2.121	151.1120 (100); 133.1013 (50); 123.1171 (30); 112.0757 (10)
dehydropipernonaline (**12**)	C_21_H_25_NO_3_	7.46	340.1905	–0.588	255.1027(90); 227.1076 (100); 179.1311(40); 161.0602 (10); 112.0759 (30)
pipernonaline (**10**)	C_21_H_27_NO_3_	8.21	342.2056	–2.251	255.1390 (10); 229.1226 (100); 220.1698 (10); 201.0354 (5)
1-(pyrrolidinyl)-11-(3′,4′-methylenedioxyphenyl)-2,4,10-undecatrien-1-one (**13**)	C_22_H_27_NO_3_	9.02	354.2059	–1.328	283.1345 (20); 255.1391 (50); 232.1705 (100); 215.1073 (50); 135.0443 (30)
piperacide (**14**)	C_22_H_29_NO_3_	9.40	356.2218	–0.703	283.1339 (40); 255.1389 (70); 234.1859 (100); 215.1074 (40); 135.1171 (40)
piperchabamide D	C_22_H_31_O_3_N	10.50	358.2370	–1.955	285.1393 (100); 257.1477 (40); 135.1170 (50)
piperundecalidine (**15**)	C_23_H_29_NO_3_	10.85	368.2213	–1.848	283.1332 (30); 255.1383 (100); 246.1850 (100); 135.0441 (55)
piperchabamide B (**11**)	C_23_H_31_NO_3_	11.74	370.2372	–1.405	285.1492(100); 257.1545 (40); 248.2019 (40); 161.0601(30); 135.0442 (70)
brachyamide A	C_24_H_31_NO_3_	12.24	382.2372	–1.361	313.1805 (30); 283.1699 (100); 135.0442 (40)
guineensine (**16**)	C_24_H_33_NO_3_	12.73	384.2525	–2.057	311.1646 (20); 283.1698 (100); 161.0598 (10); 135.0442 (30)
13-(1,3-benzodioxol-5-yl)-1-(1-piperidinyl)-2,4,12-tridecatrien-1-one	C_25_H_33_NO_3_	13.98	396.2526	–1.768	311.1646 (20); 283.1697 (100); 135.0442 (40)
*N*-isobutyl-2,4,10-hexadecatrienamide	C_20_H_35_NO	14.09	306.2786	–1.800	233.1911 (100); 205.1961 (40)
1-(1-piperidinyl)-2,4,10-hexadecatrien-1-one	C_21_H_35_ON	15.79	318.2784	–2.203	233.1910 (20); 112.0758 (100)
piperolein B	C_21_H_29_NO_3_	16.71	344.2219	–0.466	259.2063 (30); 231.2115 (100); 112.0757 (5);
*N*-isobutyl-hexadeca-2,4-dienamide	C_20_H_37_ON	16.78	308.2943	–1.756	265.4167 (10); 235.2061 (100); 182.1539 (30)
*N*-isobutyl-2,4,12-octadecatrienamide	C_22_H_39_NO	17.42	334.3100	–1.231	261.2218 (100); 233.2266 (90)
*N*-isobutyl-2,4,10,12-Octadecatetraenamide	C_22_H_37_NO	18.10	332.2942	–1.810	261.2218 18 (100); 233.2277 (90)
1-(1-piperidinyl)-2,4,12-octadecatrien-1-one	C_23_H_39_NO	18.94	346.3097	–2.083	261.2221(100); 233.2267 (40); 112.0759 (60)
pipericine	C_22_H_41_NO	19.68	336.3256	–1.580	263.2369 (100); 234.0333 (50); 157.7024 (40)
1-(piperidinyl)-2,4-octadecadien-1-one	C_23_H_41_NO	19.85	348.3257	–1.095	264.2330 (20); 236.2014 (5); 112.0758 (100)
*N*-isobutyl-2,4,14-eicosatrienamide	C_24_H_43_NO	20.09	362.3407	–2.957	289.2535 (100); 261.2585 (20)
1-(1-pyrrolidinyl)-2,4,15-eicosatrien-1-one (**22**)	C_24_H_41_NO	20.94	360.3257	–1.142	289.2533 (100); 262.2183 (20)
pipercitine	C_23_H_43_NO	21.41	350.3412	–1.403	264.2398 (90); 212.1963 (100)
1-(piperidinyl)-2,4,14-eicosatrien-1-one (**21**)	C_25_H_43_NO	21.50	374.3412	–1.473	318.28 (20); 289.2535 (100); 261.2588 (10)
1-(piperidinyl)-2,4-eicosadien-1-one	C_25_H_45_NO	22.50	376.3568	–1.545	291.2700 (100); 112.0760 (40)

**Figure 2:**
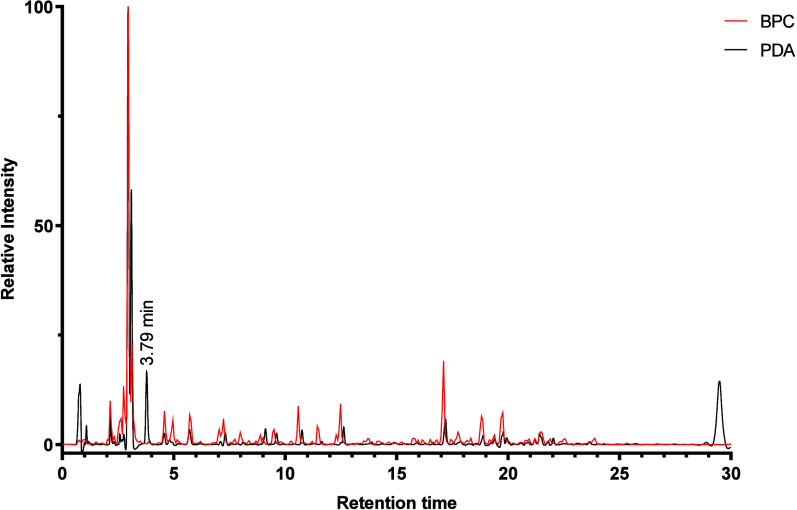
Overlay of BPC (red) and PDA (black) chromatograms of the *P. borbonense* acetonic extract.

Piperamides, the most abundant class of metabolites present in the extract, include two main subclasses, terminating, respectively, with a 1,3-benzodioxol-5-yl- (as deduced from FBMN analysis) or with a methyl group. The positive ion mode used for the LC-MS^2^ experiment was well suited for the analysis of piperamides, but the LC-UV analysis showed an interesting pattern (Figure 2). In particular, as shown in Figure 2, the photo diode array (PDA) chromatogram included several additional peaks compared to the LC-MS base peak chromatogram (BPC), suggesting the presence of additional metabolites. When LC-UV-MS^2^ analysis was performed in negative mode, the BPC resulted flat, suggesting that these metabolites are not ionizing and cannot be detected in positive or in negative mode.

### 3.3 Chromatographic Purification and GC-MS Quantification of Sesamin

In order to carry out a comprehensive and detailed analysis of the secondary metabolites present in the acetone extract of *P. borbonense* berries, chromatographic purification was needed. To this aim, the acetone extract (about 5 g) was purified by MPLC as described in detail in the Materials and Methods Section. Piperamides were, as expected, the most abundant class of metabolites present in the extract, but terpenes and lignans were also largely represented. In fact, this first purification step yielded, as major compounds, p-menth-5-en-1,2-diol (**23**, 73 mg),^19^ a dihydroxylated monoterpene never reported before in the *Piper* genus, piperine (**6**, 495.6 mg),^20^ and sesamin (**34**, 221.8 mg),^21^ while the other fractions needed further purification.

**Figure 3:**
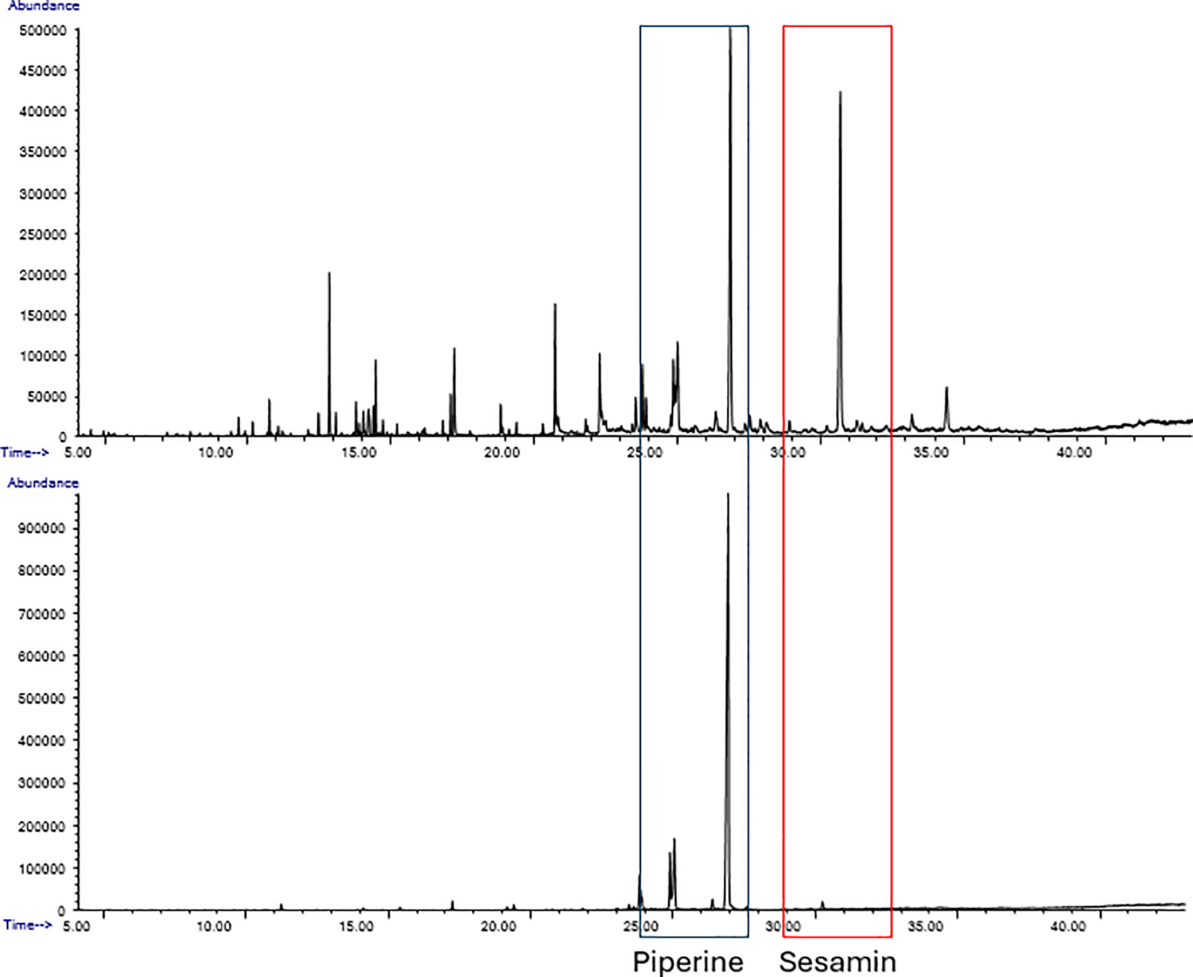
GC-MS chromatograms of *P. borbonense* (upper) and *P. nigrum* (lower) acetone extracts.

The unexpected relative abundance of sesamin, Rt 3.79 in the PDA chromatogram (see Figure 2), prompted us to carry out a quantitative analysis. The measurement of sesamin and of the main piperine isomers in the extract of *P. borbonense* was carried out via GC-MS analysis (Figure 3).

Sesamin and a mixture of piperine isomers were used as external standards, each at concentrations of 12.5, 25, 50, 100, and 200 ppm. The calibration curve demonstrated a high coefficient of determination (*R*^2^ = 0.9986–0.9993), indicating the reliability of the method. Due to their structural similarity, piperine was quantified as the sum of all stereoisomers, which eluted at retention times of 24.89, 25.04, and 26.88 min (Figure 3). The results confirmed a very high content of sesamin (152 mg/g) in the extract of *P. borbonense*, comparable with the content of piperine isomers (233 mg/g). Despite this high concentration, sesamin has never been reported before from *P. borbonense*. Interestingly, this lignan was not detectable in our samples of *P. nigrum* (Figure 3), where only small amounts had occasionally been reported.^22,23^ The relatively high concentration of sesamin had probably been missed in earlier studies of *P. borbonense* due to the almost exclusive use of LC–ESI-MS/MS for the quantitative determination of its metabolites.

According to recent studies conducted on sesame oil, where this lignan is found in high concentrations, sesamin can impact aroma^24^ directly and also indirectly, because it can affect flavor formation and improve sensory perception by interfering with lipid oxidation.^25^ Moreover, sesamin content can influence the processing steps of the biomass, but it has never been taken into account in the previous studies on the processing of *P. borbonense*.^26^

### 3.4 Isolation and Characterization of *P. borbonense* Secondary Metabolites

**Figure 4:**
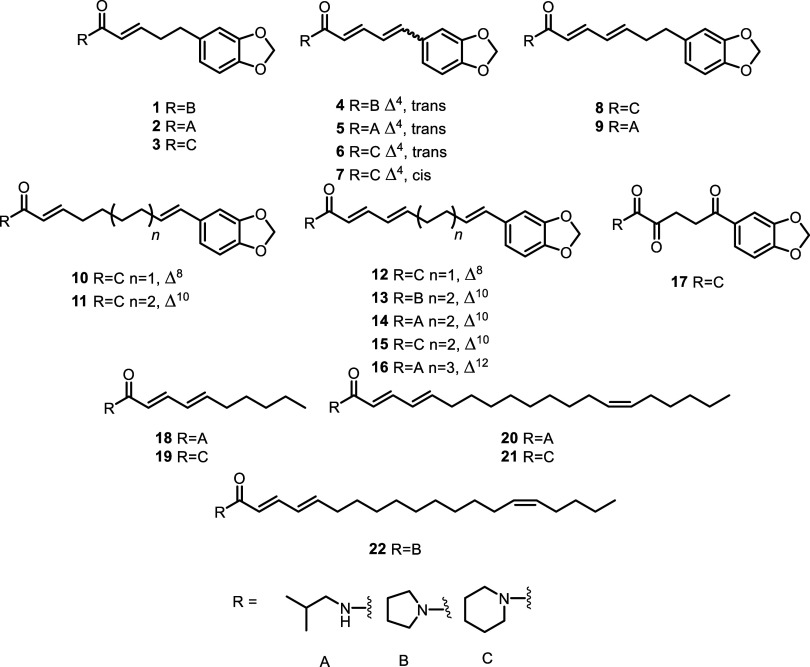
Piperamides isolated from *P. borbonense*.

Several piperamides, previously annotated via LC-MSMS were also isolated and unambiguously identified (Figure 4), generally comparing their ^1^H NMR profile with that reported in the literature. Along with the main compound piperine (**6**), the benzodioxole piperamides 4,5-dihydropiperyline (**1**),^27^ dehydropiperlonguminin (**2**),^28^ piperanine (**3**),^20^ piperiline (**4**),^29^ piperlonguminin (**5**),^20^ isochavine (**7**),^30^ piperdardine (**8**),^31^ chingchengenamide A (**9**),^32^ pipernonaline (**10**),^20^ piperchabamide B (**11**),^33^ dehydropipernonaline (**12**),^34^ (2E,4E,10E)-11-(1,3-benzodioxol-5-yl)-1-(1-pyrrolidinyl)-2,4,10-undecatrien-1-one (**13**),^35^ piperacide (**14**),^36^ piperundecalide (**15**),^37^ guineesine (**16**),^20^ and piperodione (**17**)^38^ were isolated. In addition, the following members of the N-linked long-chain fatty acids class were obtained (Figure 3): pellitorine (**18**),^39^ neopellitorine B (**19**),^32^ N-isobutyl-2E,4E,14Z-eicosatrienamide (**20**),^20^ 1-(piperidinyl)-2,4,14-eicosatrien-1-one (**21**), and 1-(1-pyrrolidinyl)-2,4,15-eicosatrien-1-one (**22**).^40^

Among the isolated piperamides, chingchengenamide A (**9**), piperacide (**14**), and neopellitorine B (**19**) have never been reported not only in *P. borbonense* but also in the common *P. nigrum*. In addition to a piperine content lower than the one of cultivated peppers, also the occurrence of a bouquet of isobutylamides (**2, 5, 9, 14, 18, 20**), a class of compounds endowed with anesthetic properties,^41^ could explain the lower pungency of *P. borbonense* compared to the common peppers of commerce (*P. nigrum* and *P. longum*), as well as its use to relieve toothache. The 14(*Z*) configuration of **20** and **21** (and 15Z of **22**) was supported by the study of fragment peaks, in accordance with their location, and of the chemical shifts of two adjacent methylene carbons (δ_C_ 27.2), in agreement with data reported in the literature.^42^ Interestingly, this configuration has been associated with a prolonged tingling sensation^40^ and, given their significant presence in *P. borbonense*, they may contribute to the peculiar taste of this spice and to its reduced pungency.

**Figure 5:**
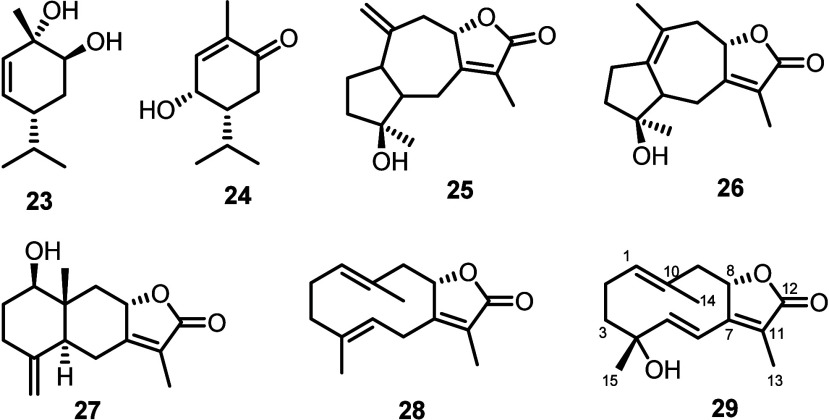
Terpenoids isolated from *P. borbonense*.

As previously stated, metabolites of the terpene class constituted a rich part of the extract. Besides the above-mentioned **23**, the related monoterpene 5β-hydroxy-p-menth-6-en-2-one (**24**)^43^ was also isolated. The sesquiterpenoids multistalactones E (**25**) and D (**26**)^44^ and neolitacumone B (**27**),^45^ all never reported before in the genus *Piper*, were also isolated, along with the previously undescribed 4-hydroxyisogelehomanolide (**29**), an analogue of the germacrenolide gelehomanloide (**28**)^46^ co-occurring in the plant (Figure 5). Noteworthy, sequiterpene lactones are common in plants from the Asteraceae and Apiaceae families but have only rarely been discovered in other plant sources, having a point-like distribution in Nature, and had never been found before in plants from the genus *Piper*.

**Figure 6:**
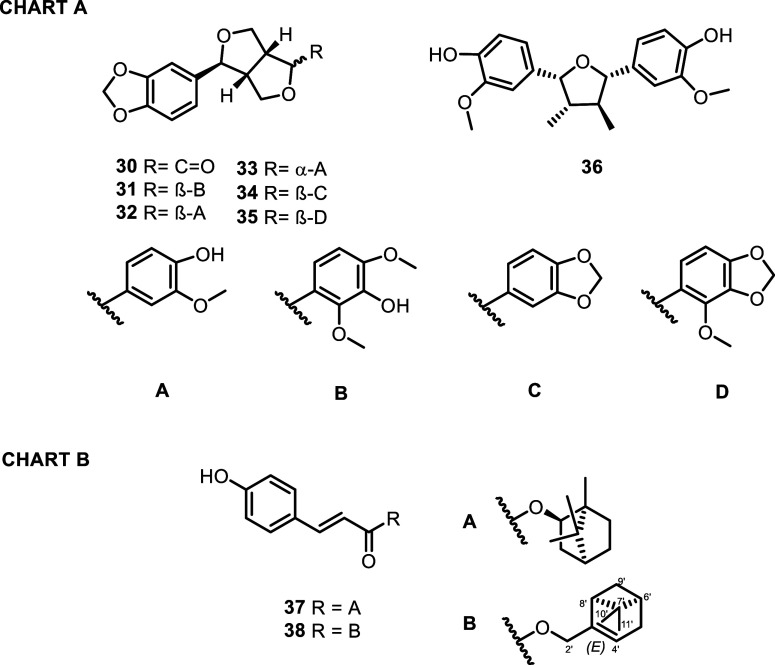
Phenols isolated from *P. borbonense*. Chart A: lignans; Chart B: hydroxycinnamic esters.

Finally, among the lignans, in addition to the abundant sesamin (**34**), 5-methoxysesamin (**35**),^47^ (+)-acuminatolide (**30**),^48^ compound **31**,^49^ piperitol (**32**),^50^ pluviatiol (**33**),^51^ and verrucosin (**36**),^51^ were also isolated. Compounds **30** and **36** had never been reported before from the genus *Piper*. Furthermore, two monoterpenyl hydroxycinnamic ester derivatives were isolated, namely, the known pressafonin A (**37**)^52^ and the myrtenoyl ester **38**, a novel compound which we have named borbonensin (Figure 6).

**Figure 7:**
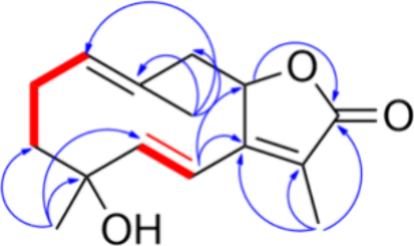
COSY (red bold) and HMBC (blue arrows) of **29**.

4-Hydroxyisogelehomanolide (**29**) was isolated as a colorless amorphous solid with HR-ESIMS *m*/*z* 249.1485, in agreement with a molecular formula (C_15_H_20_O_3_), implying six degrees of unsaturation. The ^1^H NMR spectrum of **29** was very similar to that of **28**, including, however, an additional oxygen atom. A close comparison of the ^1^H NMR spectra of the two compounds showed that the most remarkable difference was a pair of mutually coupled olefinic protons resonating at δ_H_ 6.10 (1H, d, *J* = 17.0 Hz) and 5.90 (1H, d, *J* = 17.0 Hz) in place of the doublet at δ_H_ 4.85 (1H, d, *J* = 12.0 Hz, H-5). This assignment was further supported by the inspection of ^13^C NMR and 2D HSQC spectra allowing the identification of 15 carbon signals including two allylic methyls (δ_C_ 16.9, C-14 and 23.9, C-2), one aliphatic methyl (δ_C_ 8.8, C-13), three methylenes (δ_C_ 23.9, C-2*;* 46.3, C-9 and 49.3, *C-*3), three olefinic methines (δ_C_ 116.3, C-5; 139.6, C-1; 152.9, C-6), three olefinic quaternary carbons (δ_C_ 121.6, C-11; 127.1, C-10; 159.2, C-7), three oxygenated carbons, including an oxygenated methine resonating at δ_C_ 77.8 (C-8), one quaternary sp*^3^* carbon *(*δ_C_ 75.5, C-4) and one quaternary sp^2^ carbon *(*δ_C_ 174.9, C-12). Analysis of the 2D NMR COSY experiments led to the definition of two spin systems, spanning from C-1 to C-3 and C-5 to C-6, and the assembly, guided by the 2D NMR HMBC cross-peaks, of the ten-membered ring of the germacrene skeleton. In particular, the diagnostic HMBC correlations between H_3_-15 with C-3, C-4, C-5 and of H-6 with C-4, C-7, C8, C-11 unambiguously assigned the position of the Δ^5^ double bond, while the cross peak between H-8 and C-12 confirmed the ring closure of the lactone moiety (Figure 7).

**Figure 8:**
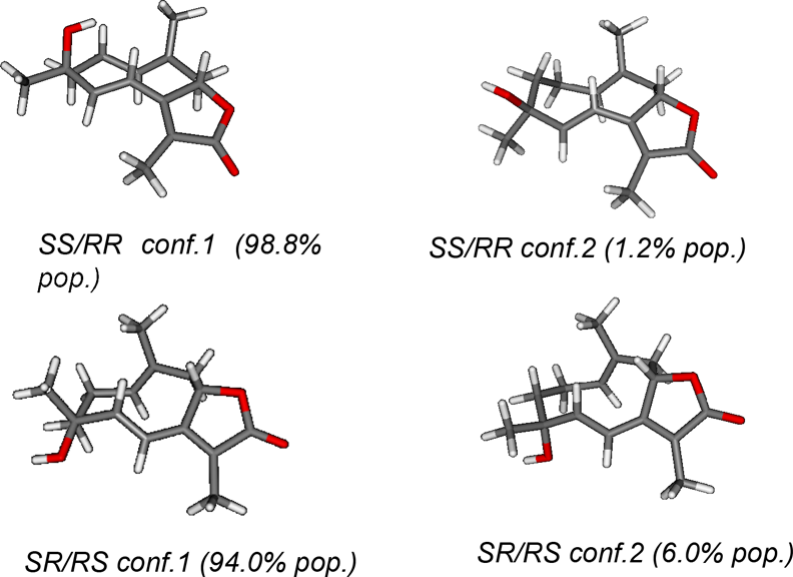
Two main conformers (and their relative population) for the two possible diastereomers of **29**.

While the E geometry of the Δ^5^ double bond could be easily deduced by the large *J*_H–H_ coupling constant, the configuration at the two stereogenic centers C-4 and C-8 remained to be determined. Since no evident through-space NMR correlation in the NOESY spectrum could be used to this aim, we decided to rely on computational calculations, reasoning that a comparison between experimental and quantum-mechanically calculated ^1^H and ^13^C NMR resonances^53^ could provide a solid indication to discriminate between the two possible relative configurations. To calculate theoretical NMR shielding constants for the two possible diastereomers (4*S*8*S*/4*R*8*R* and 4*R*8*S*/4*S*8*R*), the structures were subjected to a geometry and energy optimization using DFT with the mPW1PW91/6–311+G (d,p) functional and basis set combination using the Gaussian09. software. The different rotamers obtained for each diastereomer were geometrically optimized at the DFT level, the relative energies of all conformations were calculated, and then, the equilibrium room-temperature Boltzmann populations were obtained. Two reasonably populated conformations were obtained for each stereoisomer, and they are reported in Figure 8.

^1^H and ^13^C NMR chemical shifts were calculated for these conformers at the same level with the GIAO (Gauge Including Atomic Orbitals) option and the mPW1PW91/6–31G(d,p) DFT method (see Supplementary Data). Using the ab initio standard free energies as weighting factors, a Boltzmann average of ^1^H and ^13^C NMR chemical shifts for any given atom was independently calculated for the two diastereomers (see Supplementary Data). The computed chemical shifts for the *RS*/*SR* diastereomer matched the experimental values of **29** better than the other diastereomer, both as corrected mean absolute errors (CMAEs) and the DP4+ probability method^54^ resulted from the most likely (see Supplementary Data).

**Figure 9:**
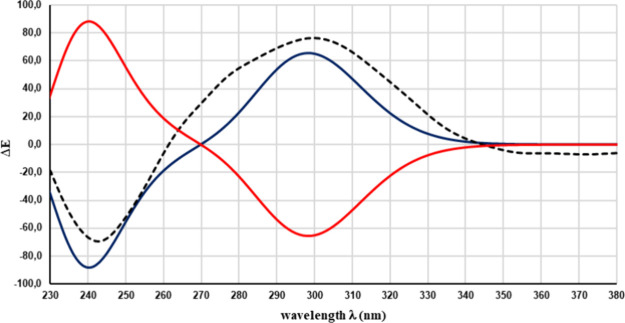
Experimental ECD spectrum of **29** (black); TDDFT-calculated curves of the 4*R*8*S*-**29** (blue) and its enantiomer (4*S*8*R*) (red).

To upgrade the relative configuration to the absolute one, TDDFT calculations were run using the functional CAM-B3LYP and the basis sets 6–31G (d,p), including at least 30 excited states in all cases and using IEF-PCM for MeOH. The rotatory strength values were summed after a Boltzmann statistical weighting, and Δε values were calculated by forming sums of Gaussian functions centered at the wavelengths of the respective electronic transitions and multiplied by the corresponding rotatory strengths. Thus, the ECD spectra for the 4*R*8*S* isomer and its enantiomer were obtained (Figure 9). The evident overlapping of the first with the experimental ECD spectrum allowed us to assign the absolute configuration of **29**.

**Figure 10:**
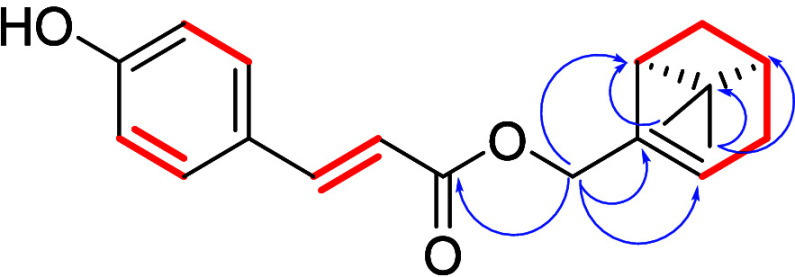
COSY (red bold) and key HMBC correlations (blue arrow) of compound **38**.

Borbonensin (**38**) exhibited HR-ESIMS peak at *m*/*z* 297.1490 [M-H]^−^, suggesting C_19_H_22_O_3_ as the molecular formula. The ^1^H NMR spectrum of **38** showed typical signals of a p-cumaroyl moiety, namely the two coupled doublets at δ_H_ 7.43 (2H, d, *J* = 8.6 Hz, H-5, H-9) and 6.83 (2H, d, *J* = 8.6 Hz, H-6, H-8), belonging to the p-disubstituted phenyl ring, and δ_H_ 7.64 (1H, d, *J* = 15.9 Hz, H-2) and 6.30 (1H, d, *J* = 15.9 Hz, H-2), belonging to the conjugated trans olefinic group in the conjugated system. In the midfield region, a signal resonating at δ_H_ 5.58 and two mutually coupled doublets at δ_H_ 4.59 and 4.56 (both 1H, d, *J* = 12.7, 1.3 Hz) were present, while the high-field region appeared crowded with partially overlapping multiplets, along with two methyl singlets at δ_H_ 1.30 and 0.85. After assigning each proton to its directly bonded carbon atom by HSQC, a combined analysis of COSY and HMBC 2D NMR spectra led to the assignment of structure **38** to this compound (Figure 10). In particular, the COSY correlations grouped the high-field signals from H-7 to H_2_*-*4 to the olefinic proton H-3 (1H, m, H-3′). The HMBC cross-peaks of H_3_-10′ and H_3_-11′ with C-5′, C-7′ and C-8′ located the unprotonated carbon at δ_C_ 37.7, bearing the two methyl groups, on a four-membered ring. The HMBC correlations of the isolated methylene (δ_H_ 4.55, 4.59) with C-2′, C-3′, and C-7′, placed the allylic oxymethylene at C-1′, thus fully defining a myrtenol unit. Finally, the HMBC cross peaks H_2_-1′/C-1 allowed us to join the hydroxycinnamic acid unit and the myrtenol moieties, confirming that **38** is an unprecedented hydroxycinnamic acid ester derivative, which we named borbonensin. Due to the positive [α]_D_ value and the absence of other stereogenic centers in the hydroxycinnamic acid residue, the myrtenol moiety can be tentatively assigned as (+)-1*S*-myrtenol. Myrtenol has been detected in the essential oils of various plants, such as *Myrtus communis*, *Aegle marmelos*, and *Rhodiola rosea*.^55^ The natural compound is commonly reported as (−)-(1*R*)-myrtenol, but since the presence of myrtenol in essential oils is commonly investigated via GC-MS, this aspect has not been sufficiently explored. Notably, in our analyses of volatile components of *P. borbonense*, both through GC-MS and HS-GC-MS, we did not detect the presence of free myrtenol. Thus, apparently, all the myrtenol production in the plant is further elaborated to borbonensin.

Capitalizing on different experimental approaches, we have characterized the phytochemical profile of voatsipérifery, the *P. borbonense* fruits, highlighting the main differences between this priced gourmet specialty and the common *P. nigrum*. In particular, the LC-MS/MS-based feature-based molecular networking analysis provided the piperamide fingerprint, supported by the subsequent purification steps, while the HS-GC-MS analysis revealed a rich and diverse terpene bouquet, as well as the presence of phenylpropanoids, a type of compound absent in *P. nigrum*. In addition, a rich variety of lignans was identified, including sesamin, whose high concentration was quantified by GC-MS. Finally, the structures of two novel compounds, a sesquiterpene lactone and a *p*-coumaroyl ester (borbonensin), were characterized in detail.

The peculiar aroma of voatsipérifery can be associated with its mono- and sesquiterpene constituents, while the rich bouquet of fatty acid amides, including those bearing a Z-olefinic group and an N-isobutyl group, as well as a high content of sesamin, could be of relevance for taste. Moreover, the high abundance of this lignan can also explain some of the bioactivities ascribed to the consumption of this spice, such as the antidiarrheal one.^56^

Overall, our investigation confirms that *P. borbonense* is endowed with a peculiar profile when compared to *P. nigrum*. However, analytical chemistry can only be a proxy of the sensory richness of an aroma: as in a musical symphony, reading the score of the individual instruments cannot convey the magic that is created when they are all played together.

## Supporting Information Available

1D and 2D NMR spectra for new compounds 4-hydroxyisogelehomanolide (**29**) and borbonensin (**38**) (PDF)
