# Supplementary material for: Analytically Unsupervised Metabolomic Profile of the Premium Malgasy Pepper Voatsipérifery (Piper borbonense): Identification of Marker Components
Source: J Agric Food Chem. 2025 Apr 11;73(16):9854–66. doi: 10.1021/acs.jafc.5c01501 (PMC12023040; doi:10.1021/acs.jafc.5c01501)
Supplement: Supplementary file 1 — jf5c01501_si_001.pdf [file jf5c01501_si_001.pdf]

Supplementary Materials

Detailed Metabolomic Profile of *Piper borbonense* Fruits, a Refined Malagasy Spice, via a Combination of Unsupervised Analytical Techniques

Elena Serino,[a] Federica Pollastro,[b] Paolo Luciano,[a] David Touboul,[c] Giovanni Appendino,[b] Giuseppina Chianese,[*,a] Orazio Taglialatela-Scafati[a]

[a]Department of Pharmacy, School of Medicine and Surgery, University of Naples Federico II, Via D. Montesano 49, 80131 Napoli, Italy

[b]Department of Pharmaceutical Sciences, University of Piemonte Orientale, 28100 Novara, Italy

[c]Laboratoire de Chimie Moléculaire (LCM), UMR 9168, CNRS, Ecole Polytechnique, Institut polytechnique Paris, Palaiseau, France

Figure S1: **^{1}H NMR spectrum of compound** 29 **($CDCl_3$, 600MHz)**.......... **2**

Figure S2: **HSQC 2D NMR spectrum of compound 29 ($CDCl_3$, 600MHz)**.......... **2**

Figure S3: **HMBC 2D NMR spectrum of compound 29 ($CDCl_3$, 600MHz)**.......... **3**

Figure S4: **COSY 2D NMR spectrum of compound 29 ($CDCl_3$, 600MHz)**.......... **3**

Figure S5: **^{1}H NMR spectrum of compound 38 ($CDCl_3$, 600MHz)**.......... **4**

Figure S6: **HSQC 2D NMR spectrum of compound 38 ($CDCl_3$, 600MHz)**.......... **4**

Figure S7: **HMBC 2D NMR spectrum of compound 38 ($CDCl_3$, 600MHz)**.......... **5**

Figure S8: **COSY 2D NMR spectrum of compound 38 ($CDCl_3$, 600MHz)**.......... **5**

Figure S9: **NOESY 2D NMR spectrum of compound 38 ($CDCl_3$, 600MHz)**.......... **6**

Table S1: **Table Conformational Analysis of the stereoisomers in the MeOH solution**.......... **7**

Table S2**: Calculated and Experimental ^{1}H NMR values for 29-a SS/RR and 29-b RS/SR**.......... **7**

Table S3**: Calculated and Experimental ^{13}C NMR values for 29-a SS/RR and 29-b RS/SR**.......... **8**

Table S4**: The calculation results of 29 stereoisomers, with mean absolute errors (MAE) values and DP4+ probabilities**.......... **8**

Figure S1: ^{1}H NMR spectrum of compound **29** ($CDCl_3$, 600MHz)

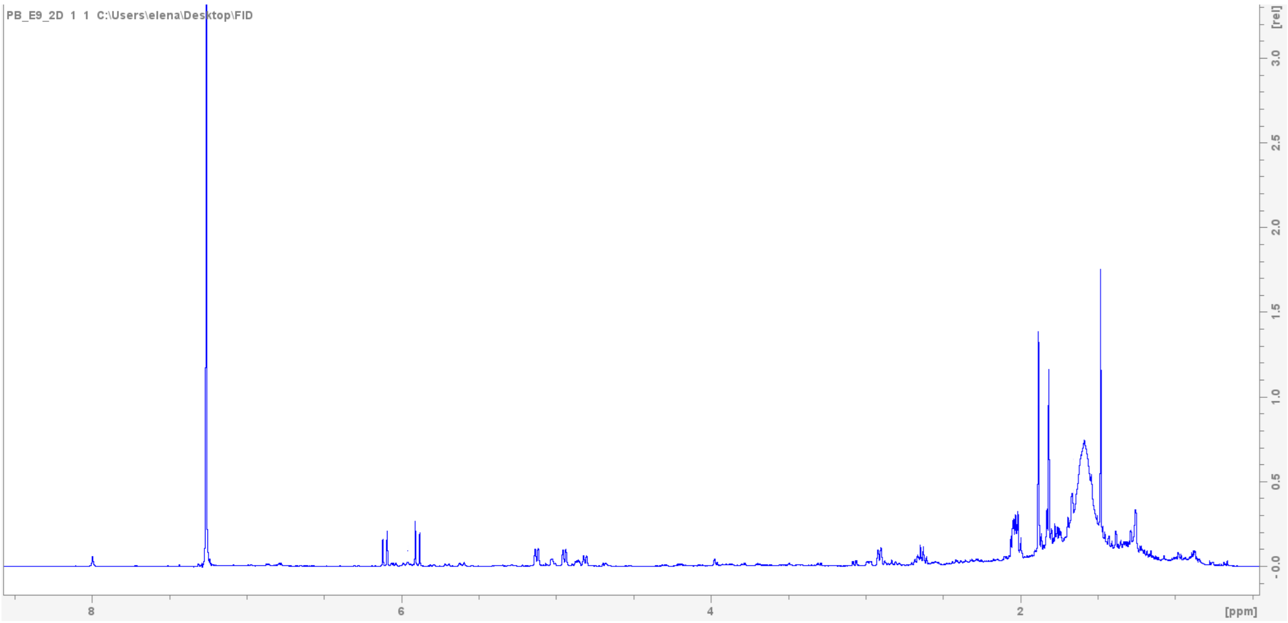

Figure S2: HSQC 2D NMR spectrum of compound **29** ($CDCl_3$, 600MHz)

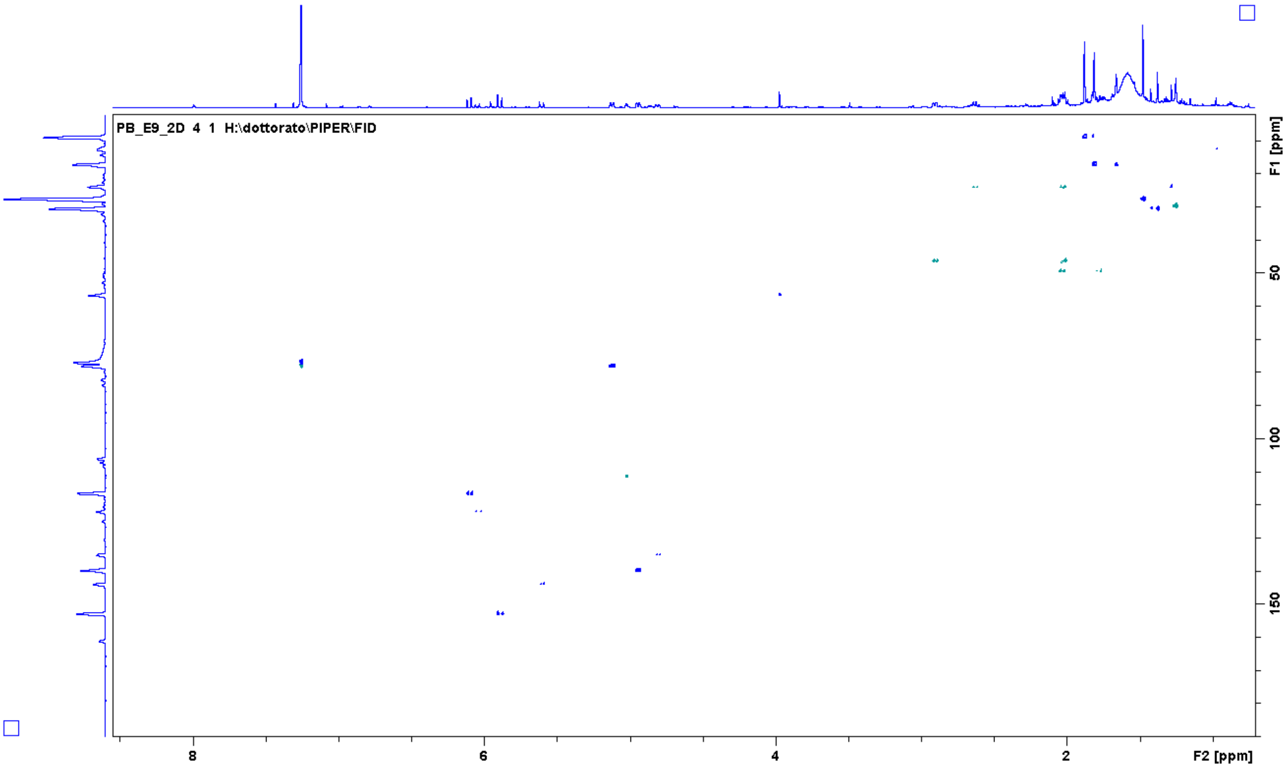

Figure S3: HMBC 2D NMR spectrum of compound **29** ($CDCl_3$, 600MHz)

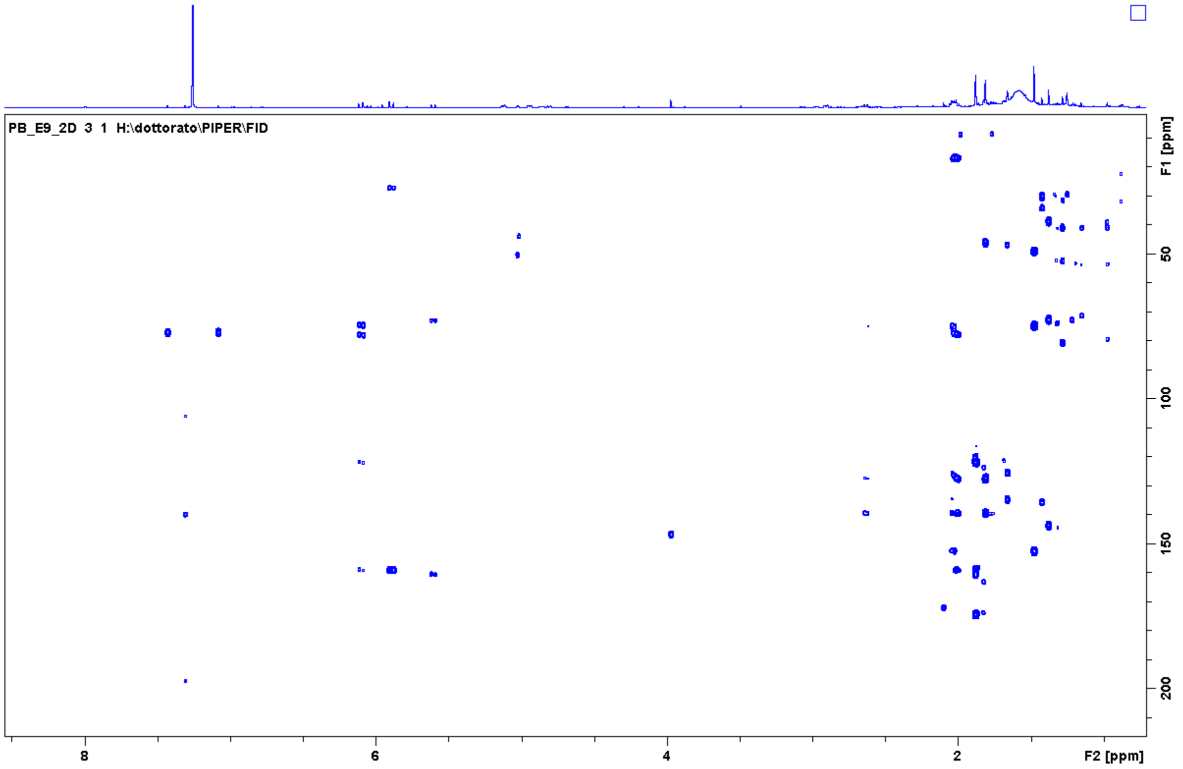

Figure S4: COSY 2D NMR spectrum of compound **29** ($CDCl_3$, 600MHz)

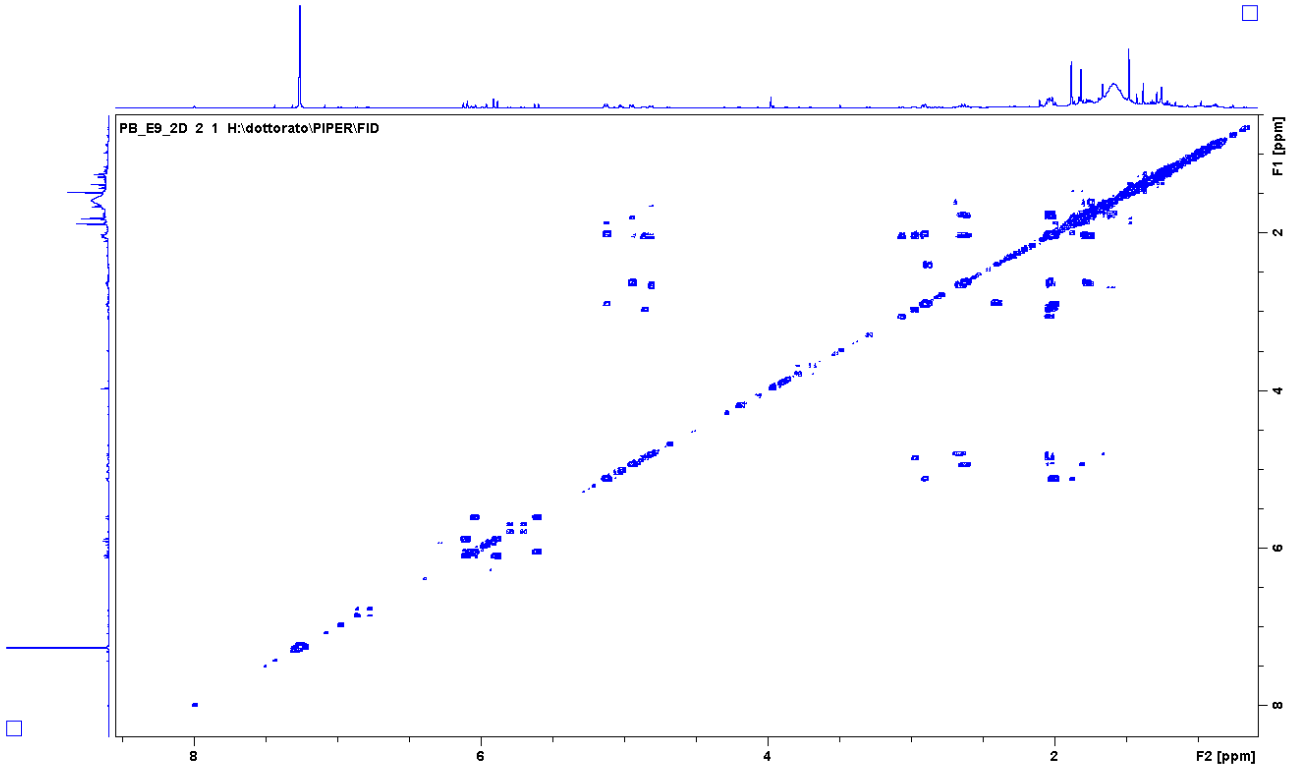

Figure S5: ^{1}H NMR spectrum of compound **38** ($CDCl_3$, 600MHz)

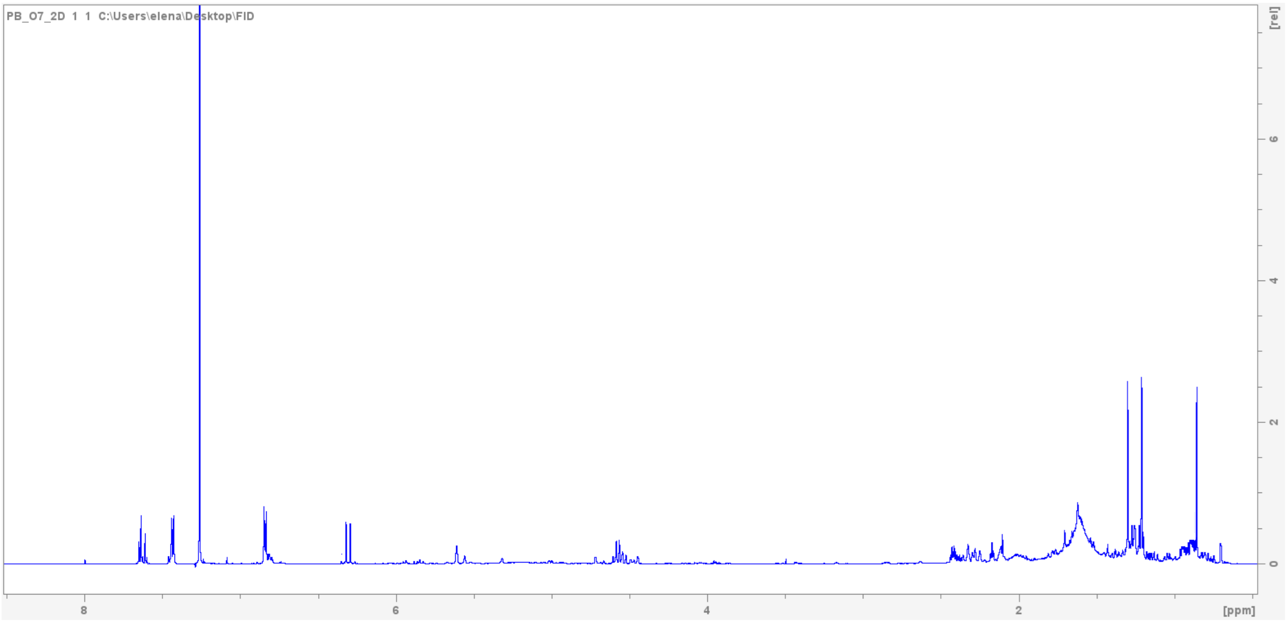

Figure S6: HSQC 2D NMR spectrum of compound **38** ($CDCl_3$, 600MHz)

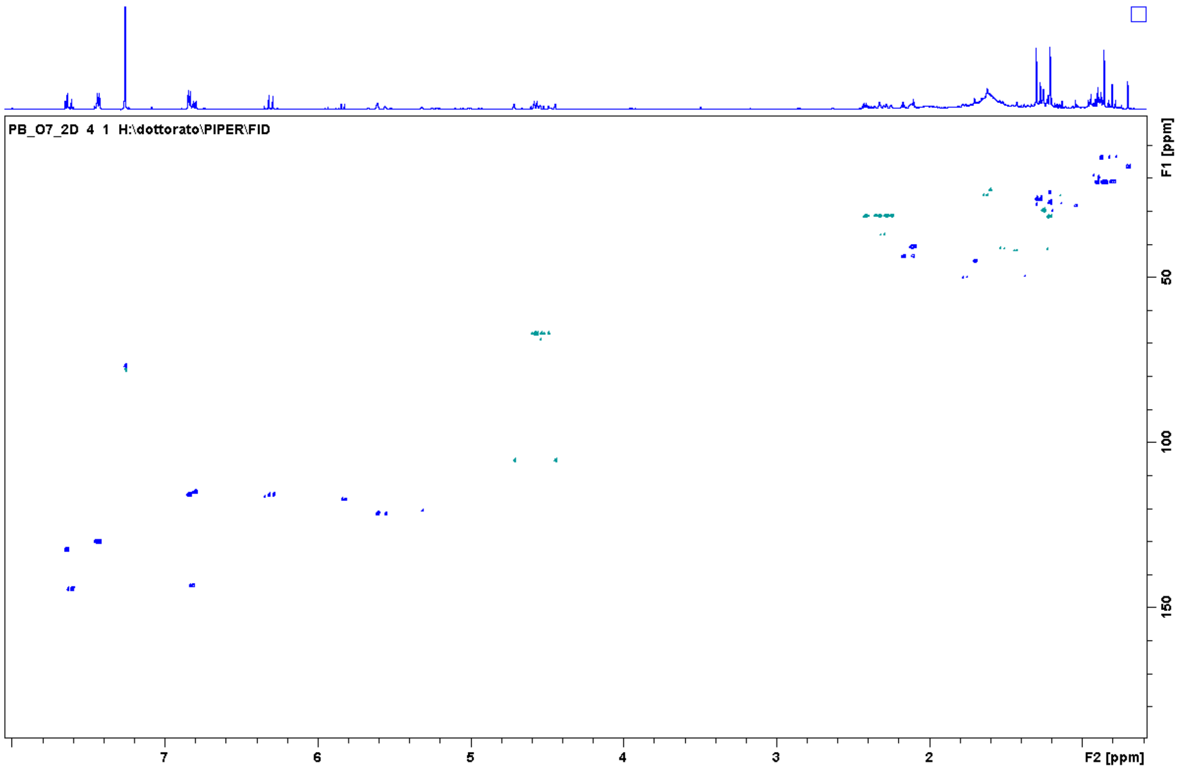

Figure S7: HMBC 2D NMR spectrum of compound **38** ($CDCl_3$, 600MHz)

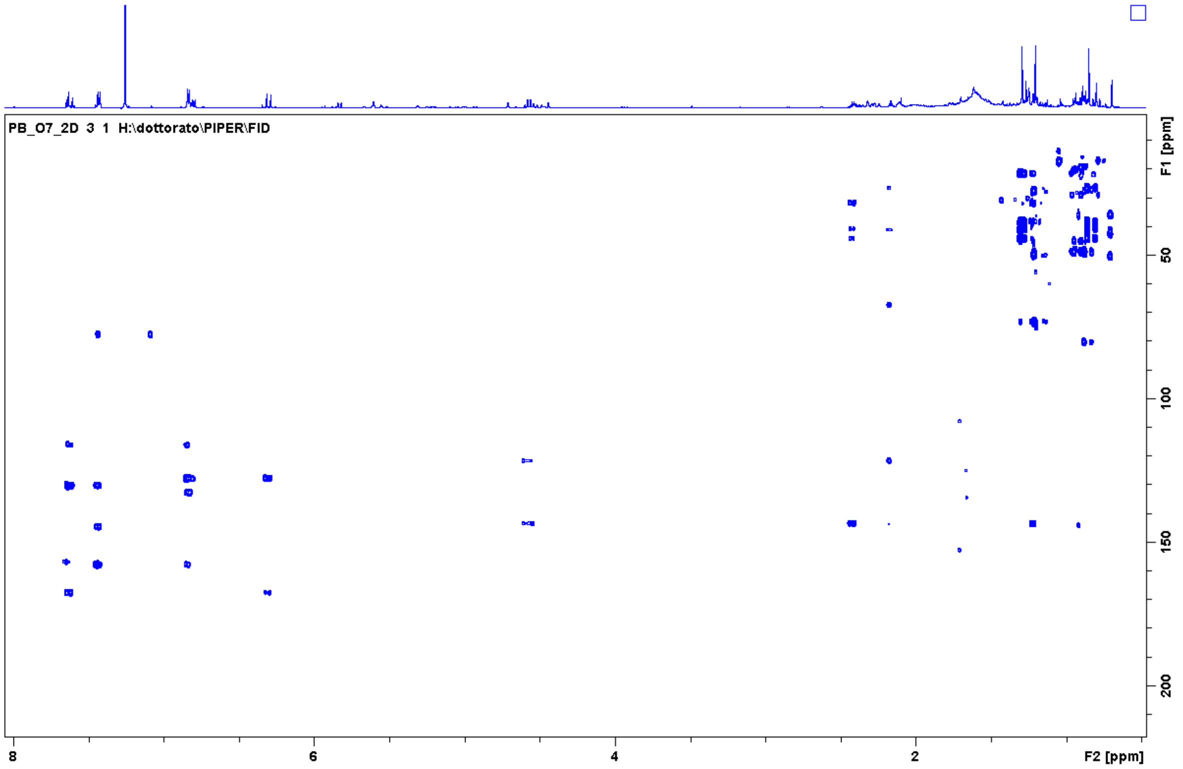

Figure S8: COSY 2D NMR spectrum of compound **38** ($CDCl_3$, 600MHz)

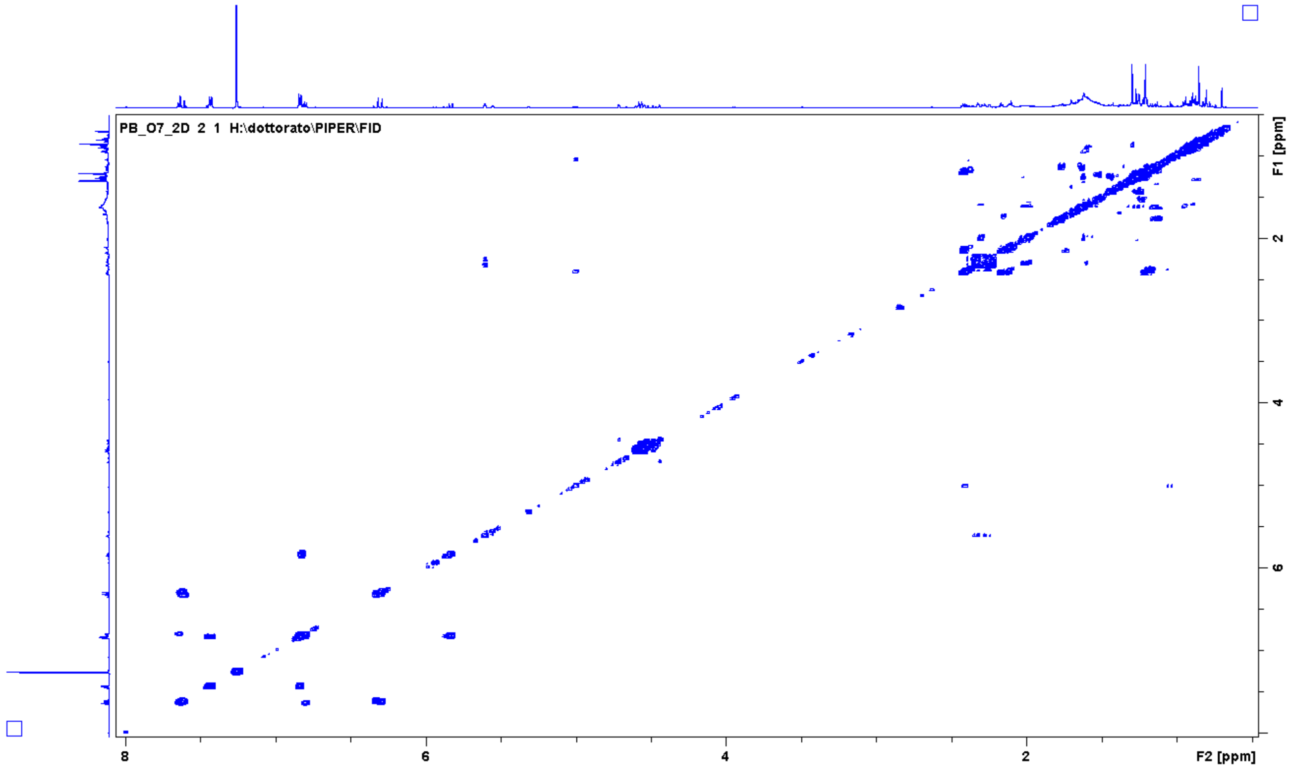

Figure S9: NOESY 2D NMR spectrum of compound **38** ($CDCl_3$, 600MHz)

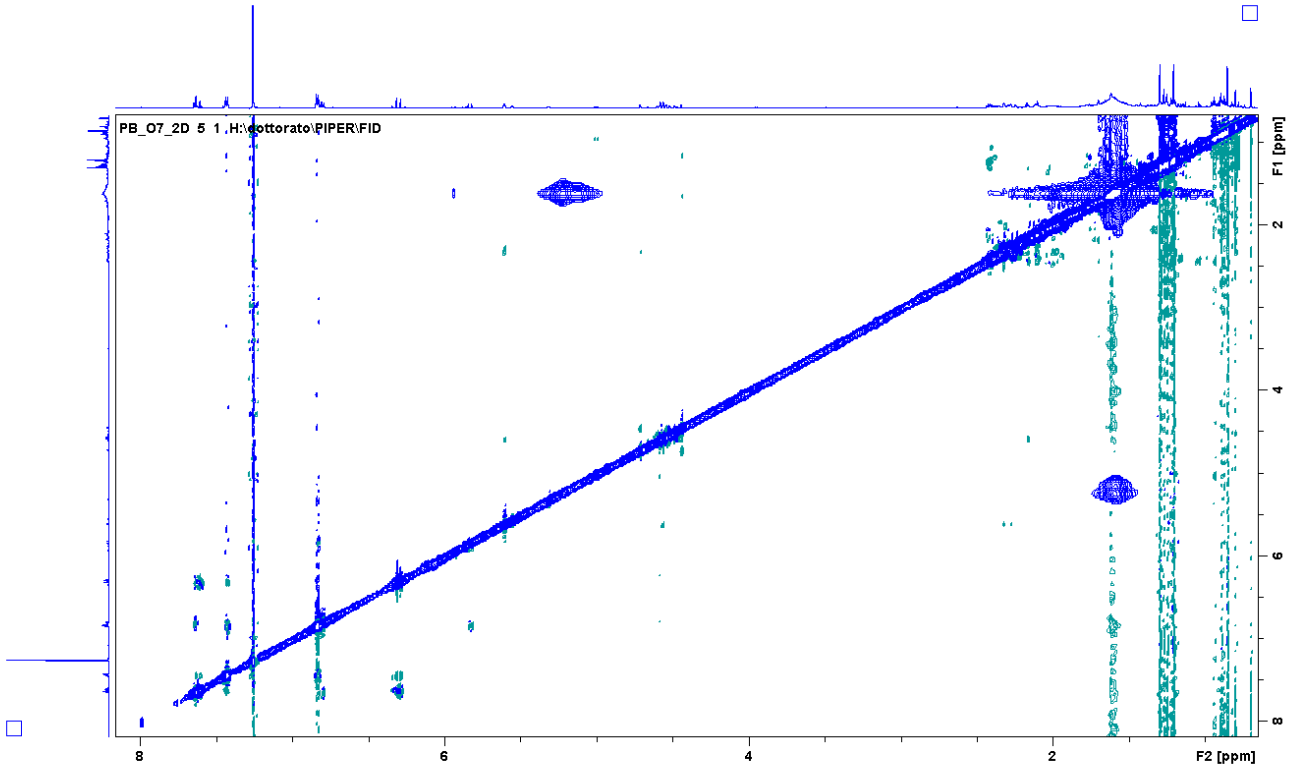

Table S1: Table Conformational Analysis of the stereoisomers in the MeOH solution

29 SS/RR		ΔE[a]	ΔG[b]	P%[c]
	29 a-1	0.00	0.00	98.8
	29 a-2	2.62	10.09	1.2
29 RS/SR				
	29 b-1	0.00	0.00	94.0
	29 b-2	1.63	6.82	6.0

[a]Relative energy (kcal/mol). [b]Relative Gibbs free energy (kcal/mol). [c]Conformational distribution calculated at the at the mpw1pw91/6-31G(d) level.

Table S2: Calculated and Experimental ^{1}H NMR values for 29-a SS/RR and 29-b RS/SR

Proton	29-a SS/RR	29-b RS/SR	Experimental
**1**	5.14	5.10	4.94
**2a**	2.31	2.33	2.63
**2b**	1.98	1.96	2.03
**3a**	1.54	1.88	2.03
**3b**	1.51	1.88	1.77
**4**	-	-	-
**5**	6.19	6.01	5.89
**6**	6.27	6.30	6.11
**7**	-	-	-
**8**	4.79	5.13	5.11
**9a**	2.84	2.70	2.9
**9b**	1.69	1.76	2.02
**10**	-	-	-
**11**	-	-	-
**12**	-	-	-
**13**	1.68	1.70	1.88
**14**	1.50	1.63	1.81
**15**	1.16	1.21	1.48

Table S3: Calculated and Experimental ^{13}C NMR values for 29-a SS/RR and 29-b RS/SR

Carbon	29-a SS	29-b RS	Experimental
**1**	130.8	138.6	139.6
**2**	24.4	26.8	23.9
**3**	37.7	52.8	49.3
**4**	72.7	72.9	75.5
**5**	147.0	149.8	152.9
**6**	116.7	113.5	116.3
**7**	159.8	156.5	159.2
**8**	74.9	76.6	77.8
**9**	47.7	45.5	46.1
**10**	125.0	125.6	127.1
**11**	118.0	119.0	121.6
**12**	167.1	167.0	174.9
**13**	10.7	10.7	8.8
**14**	17.7	17.1	16.9
**15**	26.7	27.0	27.5

Table S4. The calculation results of **29** stereoisomers, with mean absolute errors (MAE) values and DP4+ probabilities

	MAE Value (ppm)		^{13}C Data DP4+ Probability		
	^{13}C MAE	^{13}C CMAE	sDP4+	uDP4+	DP4+
**29-a SS/RR**	3.50	2.60	0.00%	0.00%	0.00%
**29-b RS/SR**	2.30	1.40	100.00%	100.00%	100.00%
	**MAE Value (ppm)**		**^{1}H Data DP4+ Probability**		
	**^{1}H MAE**	**^{1}H CMAE**	**sDP4+**	**uDP4+**	**DP4+**
**29-a SS/RR**	0.20	0.70	0.88	0.00%	0.00%
**29-b RS/SR**	0.10	0.20	99.12	100.00%	100.00%
